# Sweet tweets! Evaluating a new approach for probability-based sampling of Twitter

**DOI:** 10.1140/epjds/s13688-022-00321-1

**Published:** 2022-02-19

**Authors:** Trent D. Buskirk, Brian P. Blakely, Adam Eck, Richard McGrath, Ravinder Singh, Youzhi Yu

**Affiliations:** Bowling Green State University, Bowling Green, USA

**Keywords:** Twitter, Probability sampling, Tweets, Social media, COVID-19, Big data, Survey research

## Abstract

As survey costs continue to rise and response rates decline, researchers are seeking more cost-effective ways to collect, analyze and process social and public opinion data. These issues have created an opportunity and interest in expanding the fit-for-purpose paradigm to include alternate sources such as passively collected sensor data and social media data. However, methods for accessing, sourcing and sampling social media data are just now being developed. In fact, there has been a small but growing body of literature focusing on comparing different Twitter data access methods through either the elaborate firehose or the free Twitter search or streaming APIs. Missing from the literature is a good understanding of how to randomly sample Tweets to produce datasets that are representative of the daily discourse, especially within geographical regions of interest, without requiring a census of all Tweets. This understanding is necessary for producing quality estimates of public opinion from social media sources such as Twitter. To address this gap, we propose and test the Velocity-Based Estimation for Sampling Tweets (VBEST) algorithm for selecting a probability based sample of tweets. We compare the performance of VBEST sample estimates to other methods of accessing Twitter through the Search API on the distribution of total Tweets as well as COVID-19 keyword incidence and frequency and find that the VBEST samples produce consistent and relatively low levels of overall bias compared to common methods of access through the Search API across many experimental conditions.

## Introduction

As survey response rates continue to decline at a time when survey costs are rising, survey researchers and social scientists alike are looking more broadly for alternative ways to measure public opinion. Social Media Platforms and the data they produce is capturing the attention of many researchers as one potential alternative not only for sample recruitment (Sibona and Walczac [22], Schneider and Harknett [20] and Burke-Garcia et al. [2]) but also for substantive estimates about the economy or health, among others (see Conrad et al. [5] and Suzer-Gurtekin et al. [23], for example). There is specific interest in the Twitter platform as it represents one of the largest such platforms that is freely accessible publically. But to properly mine these data for their full potential within the survey and social sciences, more work is needed to better understand how to systematically and consistently access these data efficiently.

Currently, there are four broad avenues researchers can use to directly access Tweets from Twitter using Application Programming Interfaces (API) including the Streaming API, the Search API, the Decahose API, and the Firehose API. Usually each of these APIs are used to access Twitter at the Tweet level (Hsieh and Murphy [12]), but access to users can also be granted with varying degrees using these APIs. There are also marked differences in how these APIs capture Tweets, how many Tweets are captured, and the costs of using them. The Streaming and Search APIs both offer free access to Twitter, albeit rate limited (i.e., imposed limits on the quantity of Tweets that can be retrieved, notably at most 18,000 Tweets (in increments up to 100) every 15 minutes, or 1.728 million Tweets per day^1^). The Streaming API accesses about a 1% sample of Tweets *prospectively* in real time, whereas the Search API gathers Tweets *retrospectively* within a 7-to-10-day time frame. In contrast, the Decahose API offers a 10% sample from the full Twitter corpus (going back through the history of Twitter), whereas the Firehose API offers access to the full Twitter corpus. However, the Decahose and Firehose APIs have costs associated with their use that can vary based on project needs and timelines and Firehose access is limited to a small set of vendors. Therefore, researchers must typically address the tradeoff between coverage and cost when accessing the Twitter APIs.

Recent research has compared the performance of samples gathered from each of these APIs with a focus on keywords, users, content, and Tweet volume (Tromble et al. [26], Morstatter et al. [18], Wang et al. [27], Pfeffer et al. [19], Kim et al. [13, 14]). Wang et al. [27] verified that the Streaming API and Decahose produces samples that are approximately 1% and 10% of the entire Twitter corpus, but Pfeffer et al. [19] provided cautionary evidence that samples from these APIs may not be random samples and may over-represent certain users or groups. Kim et al. [14] reported differences across the Streaming and Search APIs noting that the Streaming API captured more irrelevant Tweets over their field period and noted impacts of rate limits on the completeness of samples retrieved using the Search API, which was also a finding in the earlier work of Tromble et al. [26].

While Twitter users, or the content they post, within a given city or country may not represent all residents of that geographic area, Twitter remains one of the primary alternate sources of data used to study and understand public opinion and societal outcomes of interest. Differences between users who are on Twitter and those who are not creates the potential for coverage error as outlined by Hsieh and Murphy [12]. Some researchers have explored methods for adjusting Twitter samples obtained from the APIs for possible coverage errors using weighting adjustments (Wang et al. [28]) assuming that the target population of interest is all residents of a city or country, for example. But if the underlying samples obtained from the Twitter APIs are not random samples, such adjustments may not be as efficient at reducing such coverage error as they could have been with random samples.

It is also important to note that not all studies define target populations in the same way. For example, Gerlitz and Reider [8] focused on understanding the so called medium-specific behavior of Twitter users such as hashtagging and retweeting. So having a method for generating random samples of Twitter users would help efficiently, and affordably, answer questions about the target population of all such Twitter users. Schober et al. [21] focused on ways to maximize so called “topic coverage” where the full corpus of Twitter content represents the target population and methods for generating random samples of the tweets themselves could prove to be an efficient, cost-effective means of representing various topics or concepts within the full Twitter corpus.

Recently, Kim et al. [13, p. 2] commented that “given the different nature of social media content, it is imperative that scholars not only evaluate existing sampling methods but also look for alternative approach[es] for sampling procedures of social media content.” Indeed, as Twitter content continues to increase in size and rate limits to accessing an ever-increasing corpus are imposed, the need for intelligent sampling methods for efficiently accessing this content in representative ways is fundamental to data quality and the research enterprise. To date there have been a small number of studies that have developed or adapted methods to generate random samples from Twitter. These studies can be grouped by whether the method focuses on the user, the tweets or a combination of the two. The bulk of the work exploring sampling from Twitter has focused on creating samples of users or a combination of users and tweets. For example, Berzofsky et al. [1] developed a method for creating probability-based samples of U.S. Twitter users that leverage the underlying format of User IDs and inferred stratum membership from selected users based on information contained in associated Tweets of the selected users. This approach leveraged sequential User IDs for Twitter in a similar way as Zhu et al. [30] leveraged content IDs to create random digit search samples for digital blog content akin to Random Digit Dialing samples of telephone numbers.

Thirumuruganathan et al. [25] also developed a sampling algorithm that is user-focused based on random walks that are informed by twitter user relationships (e.g. follow/follower) called Microblog-Analyzer. This algorithm generates samples based on Twitter user timelines to estimate aggregate measures for both user and tweet related outcomes (e.g. number of users who posted tweets including “Masks” or the overall frequency of tweets that contain “Vaccine”). This approach aimed to generate random samples that could optimize estimation within platforms, such as Twitter, that have constraints on the number of queries a user can request within a given time frame. One aspect of this algorithm that is worth mentioning is the reliance on specific keywords identified *a priori* to sampling to identify the users to be included potentially within the sample. Thus, estimates about the number of tweets containing the keyword “Covid” would be derived from a sample that relied on specifying the keyword “Covid” at the time the Microblog-Analyzer was launched. If you are concerned about the actual prevalence of “Covid” on Twitter but don’t include the keyword “Rona” this approach may actually underestimate the overall prevelance as younger people tend to refer to Covid as “Rona.” Such an error has been referred to as “specification error” in the Total Twitter Error framework as described by Hsieh and Murphy [12] and is likely a real risk for any sampling method that relies specifically on the identification of keywords for creating samples from Twitter. Moreover, we note that while this method can produce fairly accurate measures of averages and counts for any specific keyword, its reliance on keyword specification at the time samples are generated means that separate samples and additional queries are required if you have multiple outcomes of interest. In this way there is a tradeoff in accuracy and efficiency that virtually every sampling method attempts to balance.

Hino and Fahey [11] used a combination approach to generate a nationally representative sample of Twitter data for Japan. Specifically, these researchers first randomly selected a set of User IDs and proceeded to gather all available Tweets from selected users using the free Search API. The researchers found that their approach yielded more Twitter content than what would have been retrieved using the Streaming API and found reasonable concordance between their accumulated sample and the Firehose on a number of metrics including topic content and volume. Hino and Fahey [11] argued that their approach focused on gathering a representative sample from the content perspective. The researchers also noted that loss of information is possible since the maximum number of Tweets that can be gathered from a single user is 3200 resulting in possible truncation of content from very active users. Suzer-Gurtekin et al. [23] also used a combination approach to generate estimates of health and social well-being that first randomly selected Tweets archived by the Decahose for a given day and second, retrieved all tweets from those users who were identified from the selected tweets in the first step. The volume of tweets for each “represented” user were used as a weighting adjustment for generating estimates from the sample of tweets. While their approach provides a weighting adjustment to create more stable estimates that can represent the full Twitter corpus on a given day, it also requires access to the Decahose which may be cost prohibitive for some researchers.

In this paper we propose and evaluate a novel method for randomly sampling Tweets that is Tweet focused and keyword agnostic and leverages the relationship between time and Tweet IDs. To our knowledge this approach is one of the first methods that focuses on Tweets and utilizes the free version of the Twitter Search API (henceforth TSAPI) in an optimal way that respects rate limits. This method provides a representative random sample of Tweets that can efficiently represent the full corpus of Tweets that are available and be used to make content related estimates such as the frequency or the total volume of tweets containing a keyword. This method produces one random sample of Tweets that can be used to estimate frequency or totals for any number of keywords of interest. We test our method over several different days within specific Metropolitan Statistical areas to show its behavior temporally and geospatially but the method can easily scale to larger intervals of time or levels of geography as well. We find that our new method has generally lower biases than current methods available from the Twitter Search API. In the remainder of the paper we will describe the algorithm in more detail in Sect. 2 and outline the details of our experimental design and evaluation metrics in Sect. 3. We present the results of our field test in Sect. 4 and discuss future work and limitations and other considerations for sampling Twitter in Sect. 5.

## The VBEST algorithm for sampling tweets

We sought to leverage the theoretical framework of probability sampling within the context of Twitter to generate samples that can represent the full corpus of tweets that are available within a given time or area. Unlike the user-based methods that require separate samples for estimating statistics about each keyword, our new method generates one representative sample that can be used to create estimates of frequency and incidence for any number of keywords of interest. The Velocity Based Estimates for Sampling Tweets (VBEST) algorithm leverages an underlying relationship between Tweet IDs and time to generate an estimate of the overall distribution of Tweets for a specified time period and general location and consists of a four-step process including:

(1) initially sampling a small collection of Tweets across a uniform sample of time, (2) an estimation step to model twitter velocities across the entire day, (3) a third step that creates a collection of Primary Sampling Units (PSUs) comprised of time intervals with equal Tweet *volume*, based on the velocities calculated in step 2, and terminates with (4) a fourth and final step that selects a probability-based sample of the these PSUs and gathers corresponding final sample queries from Twitter to obtain the VBEST final sample of Tweets.

Alternatively, if we did not include steps 2–4 and simply sampled all Tweets uniformly across time, it would instead result in a biased sample. That is, Twitter activity fluctuates through the day so that some periods of time are less active than others (citation). Uniform sampling over time would be biased to collecting more Tweets during periods of low activity than high, hence not all Tweets would have an equal probability of selection and the resulting sample would not be representative of all activity. The addition of Steps 2–4 directly address this source of bias by leveraging a sampling framework to which we can apply probability based methods for selecting more representative and less biased samples of Tweets. We discuss each of the steps in the VBEST algorithm below.

### VBEST Step 1a: time point selection

**Figure 1:**
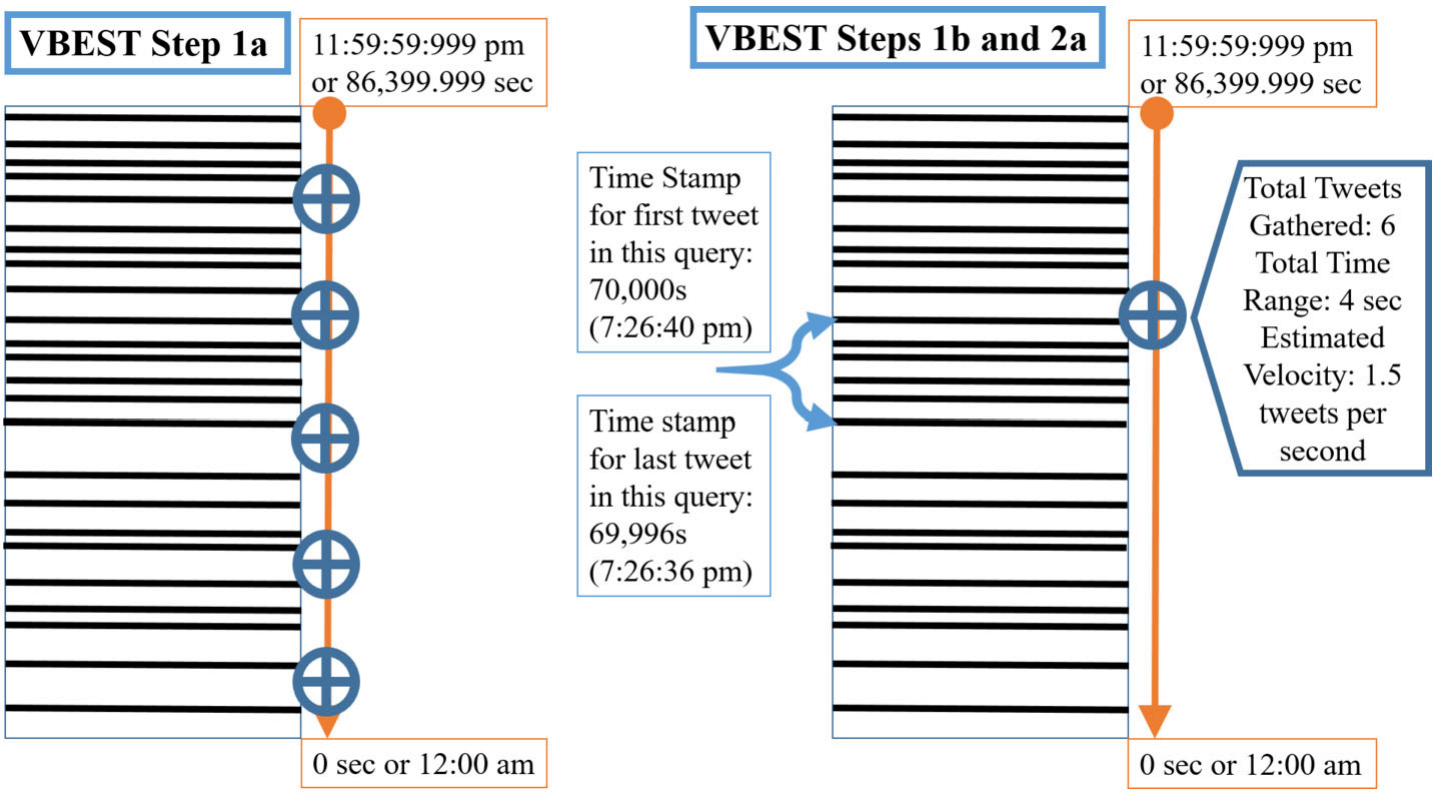
Visual depiction of the first several steps of the VBEST algorithm.: Illustration of the initial time point selection (Step 1a) as well as querying Twitter at selected time points (Step 1b) and computing Tweet Velocities (Step 2a)

In order to generate estimates of twitter volume from a given area and date, we first take a systematic sample of 48 time points by selecting a random time point within the last 30 minutes of the day (from 11:30 to 11:59:999 pm of the desired day) and then sequentially subtract 30 minutes to form a sequence of 48 systematically selected time points.^2^ We begin at the end of the day (represented by the inclusive upper bound of 86,399.999 seconds as Twitter time stamps are recorded up to the millisecond) and work our way towards the start of the day (represented as the inclusive lower bound of 0 seconds) to be consistent with the mechanism Twitter uses to store and process queries for Tweets. Specifically, as illustrated in Fig. 1, Twitter stores Tweets from a given day as a corpus that stacks Tweets from most recent downward so the last Tweet of a given day (whose time posting in seconds would be up to and including 86,399.9999) would be on the top of the corpus stack, while the first Tweet of the day occurring somewhere within the 0th second of the day onward (i.e. from 12:00 midnight or later) would be on the bottom of the corpus stack.

### VBEST Step 1b: initial tweet sample

From each of the randomly selected time points identified in Step 1a across a given day of interest, we submit two queries^3^ to the TSAPI, where each query returns a maximum of 100 Tweets with a posting time no later than a given time point. In order to ensure that the Tweets returned by each query are posted to Twitter no later than the respective time point, we converted the time point to a synthetic Tweet ID using the 64-bit representation of Tweet IDs that express time in milliseconds as described in more detail by Pfeffer et al. [19].^4^ In particular, Pfeffer et al. (2018) document that the first 42 bits of a 64-bit representation of a Tweet ID represent a date and timestamp down to the milliseconds. The remaining 22 bits represent Twitter administrative information that is not related to the timestamp. Following this relationship, we create synthetic Tweet IDs^5^ which represent the last Tweet that theoretically could have been sent before each time point, down to the millisecond. This is an important step since these synthetic Tweet IDs now index different locations in the stack of Tweets illustrated in Fig. 1, enabling us to query for the Tweets directly below that index location representing those Tweets sent immediately before the corresponding time point.

The operationalization of this step is similar to the approach of Zhu et al. [30] who leveraged a simple relationship between blog posts and blog ids. While the density of actual tweets to the total number of possible Tweet IDs is likely too low to apply their method here, we are making use of the chronological nature of Tweet IDs to time and the fact that queries made to the TSAPI can be bounded by Tweet IDs which essentially allows us to bound the query down to the nano-second. This step is also similar in spirit to the work of Dalvi et al. [6] who also address the problem of creating estimates from an unknown data set (like our full Twitter corpus) by randomly querying an API. In their work, they focus on data with a geospatial component and leverage that spatial information to determine where to query the API. We take a similar approach using temporal information instead of geospatial information. Interestingly, Dalvi et al. [6] propose that understanding the density of data points in the unknown data set affords more accurate population estimates, for which they rely on external information (e.g., density estimates of where people live geospatially). In the next step of our method, we use the Tweets collected in this step to create analogous density estimates (Tweet volumes) from the API itself without requiring external information which will be leveraged in Step 3.

### VBEST Step 2a: computation of tweet velocities from initial Twitter sample

The backbone of our sampling approach relies on our ability to estimate the unknown distribution of Tweet volume in a day. Estimating this unknown distribution, in the context of Twitter, proves to be challenging due to the imposed rate limits that significantly reduce the number of samples one can take. We introduce a volume estimator which is built *around* the imposed rate limitations, denoted as Tweet Velocity. When a query is submitted to the TSAPI, a collection of up to 100 Tweets is returned. The Tweet Velocity is simply the number of Tweets returned from a single query divided by the time spanned between the first and the last Tweets returned. This calculation results in a measure of Tweets per second for a single query, which gives us insight into the slope of the underlying volume distribution at a given time point. Combining this estimation method with our ability to sample Tweets at any given time in a day (as we will discuss), we can then estimate the underlying distribution from an entire day using the *least* resources possible.

Using the returned Tweets from the queries for each of the respective time points in the initial sample, we estimate Tweet velocity using the collection of all Tweets obtained from the two queries we submitted to the TSAPI for each of the initial time points (e.g. the total number of Tweets gathered from the two queries made at a given time point divided by the time spanned to collect Tweets from both queries). The initial sample of time points, the corresponding Twitter queries, and the calculation of the velocity estimates is illustrated in the right side of Fig. 1.

### VBEST Step 2b: estimating the Twitter velocity curve

**Figure 2:**
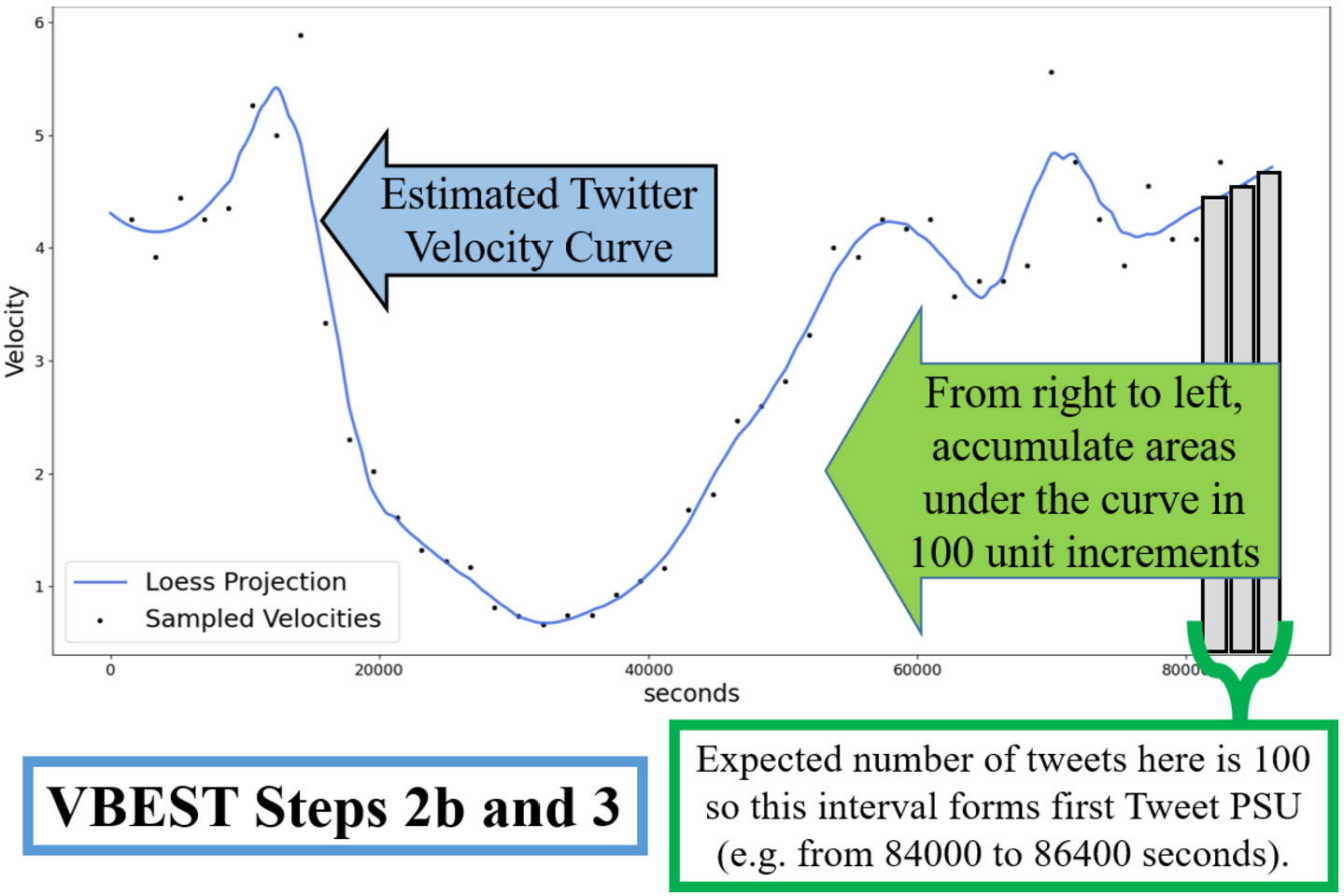
Plot of Tweet velocities and estimates the Twitter Velocity Curve (Step 2b), as well as creation of Tweet PSUs from estimated Twitter Velocity Curve (Step 3)

Using the 48 velocity estimates computed in Step 2a, a daily Twitter Velocity Curve is estimated by applying locally estimated scatterplot smoothing (LOESS) models using 2nd degree polynomials (Cleveland and Devlin [4], Cleveland [3]). The span parameter for the LOESS model was tuned across a grid ranging from 0.1 to 0.6 using 3-fold cross validation. We selected 3-fold cross validation as the total number of data points used in these models was a factor of 3 (e.g. 48 total points). An example of a Twitter Velocity Curve computed from the Tweet velocities is shown in Fig. 2.

The critical part of this step in the VBEST algorithm involves the use of some type of curve estimation or smoothing method to facilitate estimation of the Twitter Velocity curve from the velocity measures derived in Step 2a. We chose LOESS as our smoothing method due to our familiarity with the method and its popularity in the R programming language (which we used for implementing this step of the method). In our pre-testing, we also found that LOESS had good performance in estimating the true Twitter volumes with an overall error that was no larger than 3 percentage points. At the same time, other approaches to smoothing functions such as the more recent wavelet transform and Fourier transform methods could also be used in this step, and a comparison of the quality of the velocity estimates achieved by each is an interesting direction for future work to possibly further improve our method.^6^

### VBEST Step 3: create primary sampling units (tweet PSUs)

The area under the estimated Twitter Velocity Curve represents our best estimate of total Twitter *volume* for any range of time during the given day of interest. Because the LOESS curve does not have a functional form, we relied on numerical estimation methods to compute the integral of the estimated Twitter velocity curve. Specifically using the best fitting LOESS model (i.e. with the optimal span value), we estimated the total area under the Twitter velocity curve for a sequence of time points between 0 and 86,400 seconds in increments of 0.01 seconds using the Trapezoidal Rule with 2 subintervals per time point. With these area computations, we then create a sampling frame of primary sampling units that are defined as consecutive time segments that we estimate will contain approximately 100 consecutive Tweets, as illustrated in Fig. 2(B). We compute these segments consecutively from the end of the day down to the beginning of the day and will refer to them as Tweet PSUs. We set 100 Tweets as the expected target for these intervals since the TSAPI accesses Tweets using queries that return up to 100 Tweets per query.

To minimize the greediness inherent in this sequential approach and thus to minimize the cases where the final PSU (with final endpoint of 0 seconds) has considerably fewer than 100 Tweets expected, we devise a tolerance check prior to PSU construction. Specifically, denoting the estimated total daily Twitter volume derived in Step 2b as ETV, we determine the PSU target size to be the integer between 96 and 104 that evenly divides the ETV. If the ETV is not a factor of one of these integers, then the PSU target size is taken to be the integer in this range with the largest remainder in the quotient of ETV and itself. The total time covered by each Tweet PSU can vary depending on expected Twitter volume (due to the varying velocities during the day: slower velocities result in longer time spans, whereas higher velocities result in shorter time spans), but each Tweet PSU has approximately the same expected number of Tweets under the Twitter Velocity Curve.

### VBEST Step 4: final sample of tweets

With a sampling frame of Tweet PSUs constructed in Step 3, several sampling approaches can be taken to select a probability-based sample from this frame. The Tweet PSUs can be viewed as clusters of approximately the same number of Tweets. Because Tweets occur throughout the day, the default sampling method would be systematic random samples of Tweet PSUs to ensure Tweets from throughout the day can be represented (Lohr [15]). Regardless of the specific sampling method (e.g. systematic sample or simple random sample of Tweet PSUs) there is a conversion step between Tweet PSU selection and the corresponding query that is issued to Twitter via the Search API. Tweet PSUs represent time segments with a lower and upper endpoint expressed in seconds. The upper endpoint for each selected Tweet PSU is converted into a synthetic Tweet ID (similar to Step 1a) that is then used as the max_id parameter in a Search API query to extract the Tweets within the selected Tweet PSU’s time span. Finally, the queries corresponding to the sampled PSUs are sent to the TSAPI, and the returned Tweets together represent the VBEST random sample of Tweets. More information about the specific content and syntax of queries is provided in Table A1.1 in Appendix 1 and discussed in Sect. 3.2. The VBEST algorithm has been implemented using a combination of R and Python code which is available through the following repository: https://github.com/bpblakely/VBEST-Algorithm.

## Comparing methods for generating Twitter samples

To evaluate the quality of samples collected by the VBEST sampling algorithm, we conducted a multifactorial field experiment that varied the sampling method and total number of queries (determining sample size) within select Metropolitan Statistical Area regions. Replicates for the experiment were generated by fielding independent samples for each combination of method and size within each of the MSAs across multiple days in our field period. Samples will be evaluated using three specific metrics aimed at estimating: (1) the incidence rate of COVID-19 related topics, and (2) the frequency of Tweets from COVID-19 related topics and (3) total volume of Tweets in each of the MSA’s. We will compare these sample estimates to values we obtain from our Twitter Firehose Vendor (TFV), Meltwater who used advanced filtering and query parameters to access incidence, frequency and total volume of Tweets from each of our 5 MSAs using the same set of principal cities and keywords we will discuss in detail in this section. We note that in order to create as comparable measures as possible to those obtained from our TFV we had to establish some post-processing steps for our samples of Tweets since the TSAPI did not have the same query parameters that were available for the Firehose API. We constructed these post-processing filters, also described in this section, using the same logic as that used in the Firehose API queries.

### Experimental factors

**Figure A1.1:**
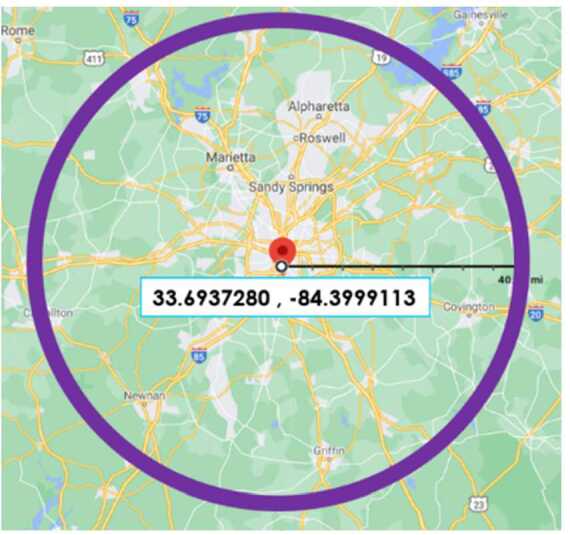
Example specification of the Atlanta–Alpharetta–Sandy Springs, Georgia MSA using the center and radius specified in Table 2. Twitter queries based on geographies in the public TSAPI require that a latitude, longitude and radius be specified rather than a place keyword or location name. Underlying Map accessed from Google Maps (https://www.google.com/maps)

1. *Metropolitan Statistical Area Regions*: We included five medium to large sized MSAs as our blocking factor including: Pittsburgh, Baltimore, Phoenix, Atlanta and Chicago. To retrieve samples using the Twitter API, we specified the latitude and longitude of the geographic center of the major primary city within each of the MSAs as determined by a query to Google Maps. We also determined a sufficiently large radius so that the circle centered at the primary city would encompass all principal cities in a given MSA region. A complete list of each of the 5 MSAs along with the principal cities, geo-coordinates used, and size of the specified radius are provided in Table 1. An example of the specification of an MSA in terms of a circular region used in the Twitter queries is provided for Atlanta in Appendix Fig. A1.1. The primary interest in our experiment is to understand broad applicability of these new sampling methods across local areas of varying sizes, so while we do not have particular interest in these specific regions, we chose them purposely based on their varying sizes and geographic shapes. In our analyses we treat them as fixed effects as they represent typical regions with varying sizes over which comparisons may be desired. We refer to the MSA factor as *region* in what follows.

**Table 1:** Listing of the 5 MSAs and Principal Cities, geo-coordinates and search radius specified for each of the Twitter queries for our experiment

Metropolitan statistical area (MSA)	Center: Lat, Long	Radius	Principal cities
Chicago-Naperville-Elgin, Illinois–Indiana–Wisconsin	41.905170, −87.624664	50 miles	Bolingbrook, IL; Chicago, IL; Des Plaines, IL; Elgin, IL; Evanston, IL; Hoffman Estates, IL; Naperville, IL; Schaumburg, IL; Skokie, IL; Gary, IN; Kenosha, WI
Atlanta–Alpharetta–Sandy Springs, Georgia	33.6937280, −84.3999113	40 miles	Alpharetta, Atlanta, Marietta, Sandy Springs, GA
Phoenix–Mesa–Chandler, Arizona	33.448400, −112.074000	53 miles	Casa Grande, Chandler, Mesa, Phoenix, Scottsdale, Tempe, AZ
Baltimore–Columbia–Towson, Maryland	39.297002, −76.676317	16 miles	Baltimore, Columbia, Towson, MD
Pittsburgh, Pennsylvania	40.437202, −79.982197	28 miles	Pittsburgh, PA
2. *Twitter Sampling/Gathering Method*: There are three options for using the TSAPI to gather Tweets including the so-called Popular, Mixed (default), and Recent methods. Because these methods are standard in the Twitter API we include them for comparison with three other approaches that include (1) Uniform sampling, which represents a systematic sample of equally spaced time points throughout the day from which to query Twitter (i.e., a systematic sample uninformed by Tweet velocities); (2) the VBEST-SYS systematic random sample proposed in Sect. 2, and (3) the VBEST-SRS simple random sample of Tweet PSUs that are constructed as part of the VBEST algorithm. The uniform, VBEST-SYS and VBEST-SRS samples make use of the recent search type option along with a max_id parameter that uses a synthetic Tweet id to constrain the time interval over which Tweets are requested. A brief description of each of the 6 sampling/gathering methods we are including in our experiment for the Method factor is provided in Table 2.

**Table 2:** Description of the 6 different methods we used in our experiment. The first three methods are possible settings for the TSAPI and the last three are new variants we are introducing and comparing in our experiment

Tweet access method	Description
1. Popular	One of three methods available for the result_type parameter in the TSAPI that returns the most popular results, as determined by Twitter, in the query.
2. Mixed	The current default method for the result_type parameter of the TSAPI: returns both “popular” and “recent” Tweets as part of the query. Popular Tweets are determined by Twitter.
3. Recent	Another option for the result_type parameter of the TSAPI that can be selected by the user in which the most recent Tweets are returned. If there are more than 100 Tweets that occurred most recently then additional queries can be submitted in sequence to obtain collections of Tweets that follow chronologically from 11:59:59:999 pm of a given day back to midnight at the beginning of that day as described in https://developer.twitter.com/en/docs/twitter-api/v1/Tweets/timelines/guides/working-with-timelines.
4. Uniform	A series of evenly spaced time points from a given day are determined a single query is submitted for each of the selected time points using the TSAPI with result_type parameter set to “recent”. For this method we randomly select a starting time point within a sampling interval determined by the number of queries desired and then determine subsequent, evenly spaced points. The identified time points are then converted to Tweet IDs and used as the max_id parameters in the TSAPI.
5. VBEST-SYS	A systematic random sample (without replacement, circular) is taken of a desired size from the universe of Tweet PSUs identified from the VBEST algorithm. The right-most endpoint of each of the Tweet PSU intervals is then used in a TSAPI query with result_type set to “recent”. One query is submitted per selected Tweet PSU.
6. VBEST-SRS	A simple random sample (without replacement) is taken of a desired size from the sampling frame of Tweet PSUs constructed from the VBEST algorithm. The right-most endpoint of each of the Tweet PSU intervals is then used in a TSAPI query with result_type set to “recent”. One query is submitted per selected Tweet PSU.

Note: For more information on Twitter Search API search options please refer to Twitter documentation available at: https://developer.twitter.com/en/docs/twitter-api/v1/Tweets/search/api-reference/get-search-Tweets.
3. *Sample Size*: To understand the impact of total queries (e.g. sample size) on sampling performance we varied the number of queries used for each method as a second experimental factor with values of 720, 540 or 360.^7^ For the first four methods described in Table 2, the total number of queries for generating samples of Tweets was exactly 720, 540 and 360. The corresponding sample sizes for both the VBEST-SYS and VBEST-SRS methods were 624, 444 and 264 queries, respectively, in order to reserve an overhead of 96 queries (e.g. 2 queries at each of the 48 initial time points) to estimate the Twitter Velocity Curve and resulting Tweet PSUs. Thus when comparing methods the net total number of queries is equivalent and sample sizes will be referred to as either 720, 540 or 360, henceforth. Neither the popular nor mixed methods had fixed sample sizes associated with them.^8^
4. *Day*: The primary time point for sampling was a given day since many studies looking to compare methods for generating samples of online content have used day as the unit of analysis (Kim et al. [13]). However, the distribution of Tweets can fluctuate from one day to another (Kim et al. [13]), so rather than selecting one or two days in the week and then selecting weekend days as is done for so called “constructed week samples”, we instead generated independent samples for each region, method and size combination for each of 38 consecutive days beginning on November 24, 2020 through December 31, 2020.^9^

### Data collection and processing

#### Twitter queries

Tweets were gathered for each of the methods within each of the MSA regions across each of the days in our field period based on varying sizes of queries. Each corresponding Twitter query was issued to the TSAPI based on a set of parameters that varied according to the sampling method, region and day. The specific parameters we used and values of them are provided in Table A1.1 in Appendix 1. All calls to the TSAPI (version 1.0) were made via the Python package Tweepy. Because the geo-location of a reTweet is assigned the value from the original Tweet and not the location of the user who reTweets and because we wanted to make comparisons of sample performance within each of the MSAs relative to benchmarks that were geographically specific, we excluded reTweets. The keyword setting used to exclude reTweets by default included all original Tweets without any additional exclusions. To allow for queries made at specific time points (as is the case for Uniform, VBEST and SRS methods) we made use of the max_id parameter to restrict gathered Tweets to be within respective time intervals as previously described. The default number of Tweets returned from any particular query is 10, but to be consistent across all sampling methods, we fixed the query size to the maximum possible value of 100 for every query. More specific information about the TSAPI parameters can be found at: https://developer.twitter.com/en/docs/twitter-api/v1/Tweets/search/api-reference/get-search-Tweets.

#### Geo-filtering cities within MSA queries

Tweets gathered across a given size of queries to the TSAPI for a given region, using a specific method on a given day, were further filtered using a geographic filter (i.e. geo-filtered) to identify all sampled/gathered Tweets that had a user location specified as one of the principal cities within the MSA listed in Table 1. This step was necessary to ensure geographic comparability between samples and information sourced from our Twitter Firehose vendor (TFV), Meltwater, who accessed Tweets directly based on the listing of principal cities given in Table 1. We note that user location fields may contain abbreviations and misspellings of a given city, and unlike the Firehose API, user location information is not translated into a city or place_id code. We note that the Firehose API has more parameter choices for geo-tag filtering at the time a query is issued, and they are based on geo-ids that take into account city name categorization. In order to maximize the sensitivity of our geo-filter, we aggregated one month of Twitter data for each region prior to our field period and identified the most frequent strings included in the location fields. This information was used to create a larger list of alternate spellings of the principal city names to use in our geo-filters. This approach allowed us to incorporate common abbreviations such as “phx” and misspellings like “Pheonix” for Phoenix, Arizona, for example. It is important to note that both the geo-filters we applied as well as those applied by our TFV made use of user location metadata and not Tweet geo-tagged metadata.

#### Filtering tweets based on keywords

In order to compare the samples retrieved from each of the methods to so called population benchmarks that we obtained via our TFV, Meltwater, we first needed to identify a common set of outcomes, akin to survey items (Suzer-Gurtekin et al. [23]). At the time our study was envisioned, the COVID-19 pandemic was a salient topic in the news and in the life of our country. We identified eight COVID-19 related topics including: Covid, Social Distancing, Working, Masks, Sanitizing, General Virus, Symptoms and Treatment. For each of these topics we identified a set of specific keywords to use in our coding of Tweets and these are provided in Table A1.2 in Appendix 1. By using a combination of standard string matching, star and near operators, we were able to construct a string filter that determined whether any of our sampled Tweets belonged to any of our eight Topics. The filtering was implemented in Python and matched the keyword filtering parameters used by our TFV. The filtering assigned a 1 or 0 per Topic to each sampled Tweet based on whether the Tweet contained at least one keyword within that Topic. We coded Tweets at the Topic level since the incidence of any one keyword could be quite small across the regions and days in our field period.

### Evaluation metrics

Incidence rates or sample proportions are common parameters of interest in many sample surveys. We evaluate how well the estimated proportion of Tweets (referred to as “incidence”) from each of our eight topics of interest compared to the values obtained from our TFV across each combination of region, method, and sample size. Here we define incidence for any of the eight topics as the proportion of geo-filtered Tweets from a given sample that contain Tweets with any of the corresponding keywords for that specific Topic.

Because we want to assess the overall performance of sampling methods and sizes, we focus on metrics that offer a summary across the various topics of interest. Such an approach has been taken by Yeager et al. [29] and more recently by Dutwin and Buskirk [7] to compare quality of non-probability samples to those of probability samples where many different outcomes are assessed by virtue of an overall summary statistic. A detailed description of each of the metrics along with formulae illustrating how they are computed is provided in Table A1.3 in Appendix 1. As in these prior studies, we use the Mean Percent Absolute Relative Bias for Incidence (MPRAB(I)) for quantifying the absolute biases across all eight topics as a summary measure of sample performance. This metric is the arithmetic mean of the eight Percent Relative Absolute Bias (PRAB) incidence measures computed for each topic for daily samples from a combination of region, method, and sample size.

Incidence rates are not always of interest for researchers who turn to Twitter data for mining tasks such as event detection, Tweet content summarization, or sentiment analyses. For these and related tasks, estimates of term or topic frequency are essential (Wang et al. [27]). To this end, we also assess topic frequency for each of our eight topics. Specifically, as detailed in Table A1.3 of Appendix 1, we use the Mean Percent Relative Absolute Bias for Topic Frequencies (MPRAB(F)) which represents the percent relative absolute biases of the frequency estimates daily for a combination of region, method and size, averaged across the eight topics.^10^ We note that the computation of frequencies for the VBEST-SRS and VBEST-SYS measures leverage the inherent cluster sampling design and sampling frame information to create an average total per sampled Tweet PSU multiplied by the total “frame size” (e.g. total number of created Tweet PSUs). But neither the Uniform nor Recent sampling methods have an associated “sampling frame” nor do they provide an estimate of the number of Tweets in a given region for a given day. So, we use a velocity-based projection measure akin to that used for VBEST-SRS and VBEST-SYS methods that is consistent with the information available for those sampling methods.^11^ While the topic frequency estimators differ across the two sets of methods (e.g. VBEST-SRS/VBEST-SYS versus Uniform/Recent), they are the most appropriate and commonly used estimators for the sampling design/collection method, and use all available information that is present within the sample and the design.

The metrics for evaluating incidence and frequencies could be applied to any particular topic or set of keywords. In our application we evaluated them specifically for the COVID-19 topics, but these topics may not be of primary interest to other researchers seeking to sample Tweets from Twitter. To this end we have included a metric that evaluates how well samples from each method and size estimate the total number of Tweets within the principal cities of an MSA region: the percent relative absolute bias (PRAB(N)), as described in Appendix 3. Similar to the frequency estimates already described, the method for estimating this topic-agnostic total is based on the VBEST sampling design for VBEST-SRS and VBEST-SYS methods and the velocity-based method for both the Uniform and Recent methods, regardless of sample size.^12^

### Analytic approach

Our primary analyses will focus on three key metrics including: MPRAB(I), MPRAB(F) and PRAB(N). For each metric we explore the relationship between it and region, method, and size using a multifactor analysis of variance model that included region, method and size as fixed effects along with Day as a random effect. The full model for each primary metric included main effects as well as two and three-factor interactions. We make comparisons of methods, sizes and regions as appropriate (e.g. either as main effects or via interactions that are significant) using Tukey’s Honest Significant Difference (HSD) post-hoc tests where the overall, experiment-wise Type I error rate was constrained to be 0.15 for each respective metric given the overall number of post-hoc comparisons that might need to be made.

## Results

In total, we collected over 112 million Tweets across the 5 MSA regions over our 38-day field period. While we sought an equal number of queries, there was some variability in the total number of Tweets gathered from each region as shown in Table 3. There is no guarantee from the TSAPI that the number of requested Tweets will be equal to the number returned for various technical reasons, and we did see some variations consistent with this limitation. But in general, we collected approximately 22.4 million Tweets in total per region across the field period. The geo-filter rates within each region varied as we expected since the initial queries had to include a circular geospatial boundary. Some regions (e.g., Atlanta) were better approximated by a circle compared to others (e.g., Baltimore), and still others had a large number of principal cities within the circular border (e.g., Chicago). Generally, we saw geo-filtering rates for our samples range from an average of approximately 83% in Chicago, 79% in Atlanta, 71% in Baltimore, 76% in Phoenix and 50% in Pittsburgh. The geo-filtering rates were consistent across the different sizes of queries for the sampling methods where queries were issued (e.g. Recent, Uniform, VBEST-SYS and VBEST-SRS). However, we did see some significant differences in the geo-filtering rates across these four methods, with Uniform usually having the lowest geo-filtering rate, VBEST-SYS and VBEST-SRS having similar geo-filtering rates and recent having the highest rates. The differences noted were quite small (usually occurring in the third or fourth decimal place) and given the sizes of samples we were collecting daily, were not practically significant. Tweets without user location metadata could not be geo-filtered and the rate at which this information was missing was quite low across our field period, as illustrated in the last column of Table 3. Generally speaking, most Tweets we captured had some information included in the user city metadata, but not all was relevant for our geo-filters (e.g. someone in Atlanta indicated their city was CandyCastle). In this case, our geo-filters would not have included those Tweets since the information on city location did not match our list of cities. So, it is possible that the geo-filter could have a higher number of false negatives as it relates to MSA inclusion. However, there is no indication that these false positive rates would be higher for one sampling method versus another but may vary overall by MSA depending on the Twitter culture for posting locations in the local regions.

**Table 3:** Distribution of sampled Tweets, geo-filtered Tweets and Tweets filtered into one our Topics by MSA and overall for our experiment. These counts represent samples gathered from all methods and sizes included in our experiment over the 38 days in our field period

Metropolitan statistical area (MSA)	Total tweets sampled	Total tweets geo-filtered into principal cities	Total tweets filtered in to one of our 8 topics	Tweets from sample missing user geography metadata
Chicago–Naperville–Elgin, Illinois–Indiana–Wisconsin	22,442,771	18,525,841	531,402	178
Atlanta–Alpharetta–Sandy Springs, Georgia	22,413,310	17,624,013	329,958	90
Phoenix–Mesa–Chandler, Arizona	22,607,714	17,191,865	640,358	60
Baltimore–Columbia–Towson, Maryland	22,435,821	16,087,382	446,228	29
Pittsburgh, Pennsylvania	22,424,971	11,099,119	393,554	309
Grand Total from All MSA Regions in the Experiment	112,324,587	80,528,220	2,341,500	666

The overall average values of our three metrics (and their standard deviations) are given by combination of region, method and size in Table 4. Of particular note is the sheer magnitude of difference between the metrics derived from samples gathered using both the Mixed and the Popular methods. In nearly all instances, the Popular method returned very few, if any, geo-filtered Tweets and among those, contained no Tweets associated with any of our topics. This resulted in constant mean percent relative absolute bias measures of 100. The Mixed method produced estimates that were slightly more varied in comparison to Popular, but overall, estimates of bias for topic incidence, frequency and MSA Tweet population size from samples using both the Mixed and Popular methods were consistently several orders of magnitude higher than any of the other methods. Given the near ubiquitous poor performance of both the Mixed and Popular methods, we excluded them in the statistical analyses comparing methods, sizes and regions for each of our three metrics in order to more readily focus on the other four methods which might be more appropriate for research inquires of Twitter.

**Table 4:** Overall averages (and standard deviations) for the three primary statistical outcomes by MSA region, size and method. The overall averages represent the arithmetic mean for the metric within a given combination of MSA Region, Size and Method across the 38-day field period for our experiment. Standard deviations are also given in parentheses

Outcome	MSA	Size and method													
		264/360				444/540				624/720				Other	
		Recent	Uniform	VBEST-SRS	VBEST-SYS	Recent	Uniform	VBEST-SRS	VBEST-SYS	Recent	Uniform	VBEST-SRS	VBEST-SYS	Mixed	Popular
*MPRAB(I)* Based on Tukey’s HSD, differences in column means^∗^ *within* a Region exceeding 2.58 in absolute value deemed significant at *α* = 0.03.	Atlanta	19.43 (6.69)	17.67 (5.66)	17.92 (6.47)	15.83 (4.92)	16.36 (5.49)	13.81 (4.63)	13.41 (3.12)	14.64 (4.65)	13.84 (4.17)	12.6 (3.54)	11.94 (3.71)	10.45 (3.11)	138.53 (88.98)	100 (0)
	Baltimore	17.24 (7.67)	14.43 (3.08)	16.01 (4.74)	13.62 (4.14)	14.11 (5.92)	12.49 (3.06)	11.28 (3.38)	10.5 (2.9)	7.99 (4.18)	10.84 (3.25)	8.89 (2.71)	8.8 (3.03)	132.02 (44.73)	100 (0)
	Chicago	14.93 (4.45)	13.59 (4.82)	15.05 (5.28)	14.15 (4.04)	12.49 (4.1)	10.47 (2.94)	11.61 (3.47)	11.36 (3.25)	10.93 (3.83)	10.3 (2.78)	9.39 (3)	9.46 (2.64)	129.94 (74.2)	100 (0)
	Phoenix	14.09 (4.59)	11.98 (3.52)	13.96 (4.1)	13.66 (3.51)	12.57 (4.02)	9.86 (2.76)	8.44 (2.53)	9.73 (3.33)	7.49 (1.85)	9.37 (3.9)	7.86 (2.08)	6.88 (2.07)	41.96 (17.09)	100 (0)
	Pittsburgh	17.18 (7.05)	15.22 (5.52)	17.18 (5.08)	15.17 (6.19)	15.12 (6.64)	12.33 (4.36)	12.07 (4.31)	11.45 (3.07)	12.8 (6.88)	10.37 (3.36)	8.96 (2.44)	9.62 (3.23)	308.86 (727.05)	100 (0)
*MPRAB(F)* Based on Tukey’s HSD, differences in column means^∗^ *within* a Region exceeding 3.70 in absolute value deemed significant at *α* = 0.03.	Atlanta	26.95 (12.25)	38.38 (4.86)	18.44 (5.58)	17.28 (4.4)	26.64 (11.3)	39.13 (5.56)	16.13 (4.15)	16.56 (5.27)	26.66 (9.55)	37.99 (4.75)	14.71 (3.73)	13.35 (3.91)	98.26 (5.1)	100 (0)
	Baltimore	33.98 (10.57)	35.2 (4.66)	17.81 (4.89)	15.48 (4.22)	26.43 (12.26)	34.07 (4.59)	14.16 (3.73)	13.86 (2.9)	10.96 (4.21)	34.62 (4.1)	12.67 (3.49)	12.46 (3.67)	94.5 (4.49)	100 (0)
	Chicago	22.3 (8.48)	41.39 (4.02)	17.97 (5.37)	17.93 (4.65)	22.4 (8.52)	42.06 (3.03)	15.93 (3.89)	16.09 (4.4)	23.3 (8.72)	42.95 (3.81)	15.03 (3.68)	14.88 (3.04)	94.85 (4.96)	100 (0)
	Phoenix	32.2 (11.03)	42.64 (3.55)	16.62 (3.94)	16.91 (5.02)	19.06 (9.21)	43.64 (3.77)	13.44 (3.1)	13.99 (3.6)	13.45 (3.41)	43.86 (3.41)	13.02 (3.34)	12.34 (3.38)	93.15 (2.05)	100 (0)
	Pittsburgh	36.73 (15.52)	30.86 (4.64)	18.33 (5.52)	16.98 (5.68)	33.51 (12.49)	29.54 (4.98)	14.55 (4.12)	13.78 (3.61)	12.44 (4.59)	30.2 (3.8)	11.94 (3.45)	12.49 (3.87)	116.39 (82.84)	100 (0)
*PRAB(N)* Based on Tukey’s HSD, differences in column means^∗^ *within* a Region exceeding 2.65 in absolute value deemed significant at *α* = 0.03.	Atlanta	19.15 (6.78)	35.65 (2.51)	7.24 (3.88)	7.12 (3.8)	19.83 (7.25)	35.6 (2.5)	7.19 (3.79)	7.19 (3.83)	21.25 (7)	35.54 (2.63)	7.23 (3.85)	7.24 (3.84)	92.07 (5.1)	100 (0)
	Baltimore	27.28 (11.22)	30.34 (3.26)	7.05 (2.73)	7.01 (2.66)	18.52 (11.24)	30.32 (3.22)	7.13 (2.66)	7.04 (2.65)	9.73 (4.63)	30.34 (3.3)	7 (2.71)	7.03 (2.65)	93.36 (1.14)	100 (0)
	Chicago	17.29 (6)	41.03 (2.24)	10.81 (2.3)	10.84 (2.29)	19.42 (6.48)	40.94 (2.22)	10.85 (2.35)	10.86 (2.32)	21.25 (6.91)	41.05 (2.23)	10.85 (2.34)	10.84 (2.3)	92.46 (1.33)	100 (0)
	Phoenix	26.02 (7.55)	43.29 (1.96)	11.34 (2.7)	11.44 (2.68)	16.16 (7.03)	43.43 (2)	11.38 (2.68)	11.34 (2.68)	15.35 (1.75)	43.35 (2.02)	11.34 (2.65)	11.35 (2.67)	92.6 (1.09)	100 (0)
	Pittsburgh	30.21 (16.12)	29.2 (3.72)	8.76 (2.72)	8.66 (2.52)	23.23 (13.45)	29.32 (3.7)	8.92 (2.48)	8.89 (2.43)	13.4 (7.19)	29.38 (3.72)	8.78 (2.55)	8.82 (2.41)	89.37 (11)	100 (0)

^∗^Note that the “Mixed” and “Popular” columns are included for reference only and were not included in any of our analyses or pairwise comparisons as described in the text.

### Topic incidence

**Figure 3:**
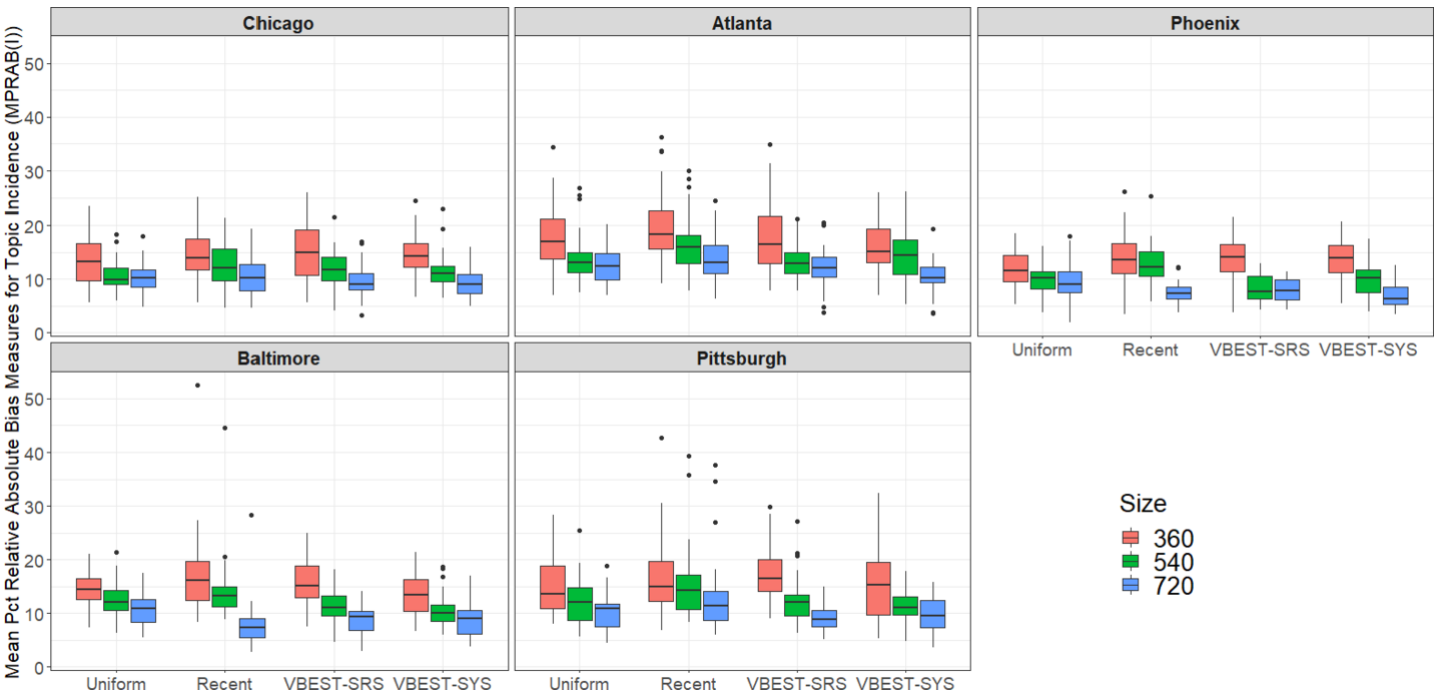
Boxplots depicting the mean percent relative absolute bias measures for topic incidence (MPRAB(I)) for each of the methods and sample sizes within each of the five MSA regions. Here we note that lower values are better

The multifactor analysis of variance model fit using daily MPRAB(I) metrics computed for samples selected from each combination of Region, Method and Size indicated a significant three-factor interaction between Method, Size and Region $(\mathrm{F}(24, 888))=2.699$; $\mbox{p-value}<0.0001$). The distribution of MPRAB(I) metrics for each Region by Method and Size are presented in Fig. 3 where we see that, across Method and Region, mean MPRAB(I) decrease with increasing Size. Within Region, Recent is usually the worst performer with the exceptions of $\mathrm{Size} =720$ in Baltimore and Phoenix. Also, VBEST-SRS and VBEST-SYS have very similar performance and are generally better than Recent and Uniform.

To explore the nature of the interaction between Region, Method and Size, we investigated the differences in MPRAB(I) means for each of the 12 Method-Size combinations (66 pair-wise comparisons) within each Region based on Tukey’s HSD procedure with a type-I error rate of 0.03 within Region and an overall experiment-wise error rate of 0.15. Specific results of these pair-wise comparisons are summarized in Table A2.1 in Appendix 2 but generally, the Recent method produced metrics that were on average larger than those derived from other samples at each of the sample sizes with few exceptions as noted. The larger values of MPRAB(I) values were more likely a result of *overestimated* incidence as generally these samples had higher rates of positive relative biases (between 45% to 65%) compared to samples from the other methods (between 30% to 50%) but this rate varied by region and to some extent sample size. For 360 Size, the other methods performed about the same as Recent with the 540 Size. This indicates a slight gain in efficiency in terms of sample effort, queries needed, and overall mean absolute relative bias for Uniform, VBEST-SRS and VBEST-SYS methods in comparison to Recent. Therefore, applying a sampling method to the “recent” result_type offered by the TSAPI is beneficial in the representation of the Tweets returned by the API, especially when the number of available queries is most limited (e.g., when trying to spread the 180 queries allowed by the TSAPI per 15 minutes across numerous geographic regions).

We also examined the standard deviation of the MPRAB(I) metrics over the 38-day period as shown in Table 4 and found that samples gathered using the Recent method had about 35% higher standard deviation in MPRAB(I) metrics across the field period compared to any of the other methods (all $\mbox{p-values} <0.005$).

### Topic frequency

Examining biases in the topic frequencies or totals gives us a way to zoom in on the performance of these sampling methods since virtually all our topics have rather low incidence relative to daily twitter activity over our field period. Since the incidence rates are rather low and use a rather large denominator (e.g. number of geo-filtered Tweets), small misses in total numbers of Tweets within associated topics may not change the low incidence rates by a discernable amount. Since the MPRAB(F) metrics use the total number of Tweets, the scale is much more sensitive to small misses in associated Tweets. Therefore, evaluating frequency rather than incidence rates focuses more carefully on the total topic recall of the sampling methods.

**Figure 4:**
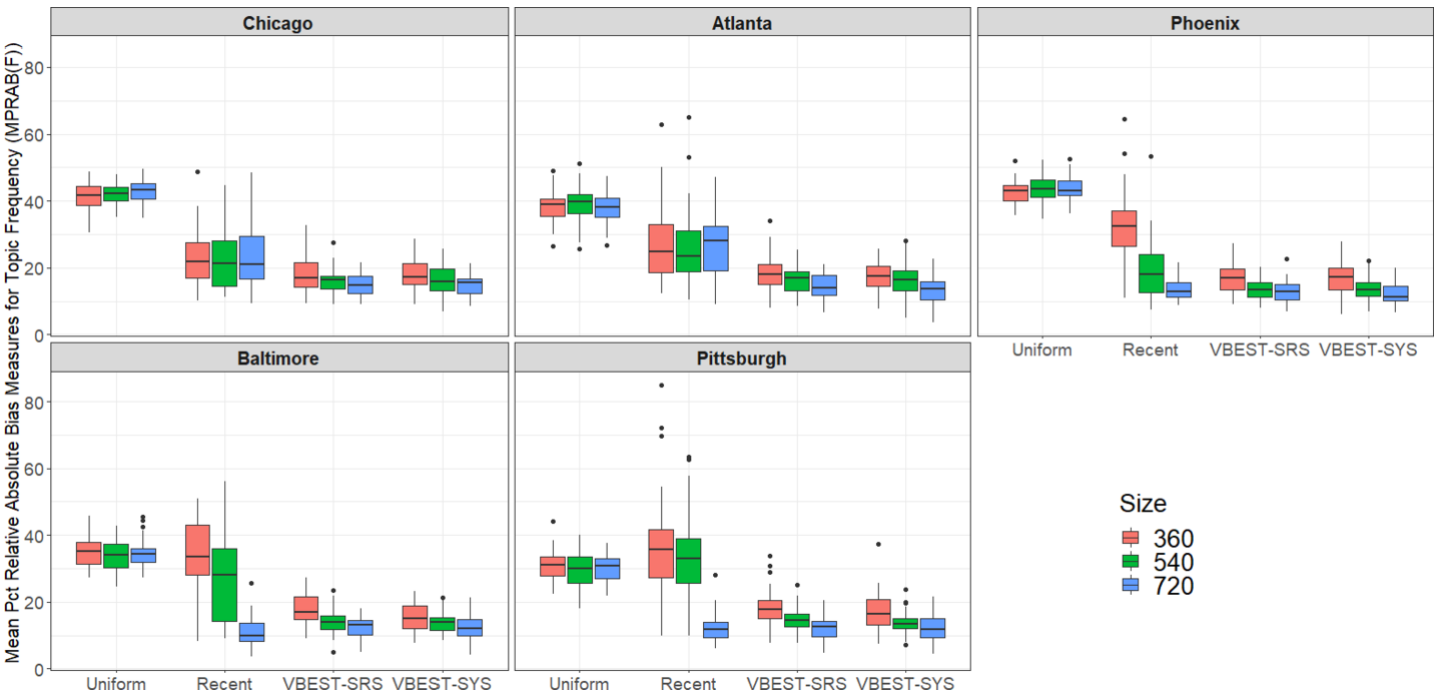
Boxplot depicting the distribution of daily MPRAB(F) metric values for topic frequencies for each Method and Size within Region. Here we note that lower values indicate smaller biases

We used a second multifactorial model to examine differences in the Mean Percent Relative Absolute Biases in Topic frequencies (MPRAB(F)). In this analysis, the total number of Tweets identified for each topic within each Region and Day were obtained from our TFV and used as the “gold standard” value in computing the MPRAB(F) metrics described in Table A1.3 in Appendix 1. The distribution of MPRAB(F) metrics across the 38-day field period are displayed in Fig. 4 by MSA Region, Method and Size. The multifactorial analysis of variance model fit to MPRAB(F) metrics indicated a significant three-factor interaction between Region, Method and Size ($\mathrm{F}(24,888)=19.34$; $\mbox{p-value}<0.0001$).

We can draw some general conclusions from Fig. 4. The mean MPRAB(F) for Uniform is about the same regardless of Size and is consistently the worst of the four Methods.

Recent is the next worst and has approximately the same performance regardless of Size in Chicago and Atlanta but improves with increasing Size in other regions. VBEST-SRS and VBEST-SYS are very similar and improve with larger Size. Table A2.2 in Appendix 2 presents more specific differences across Method and Size within each of the regions based on a series of post-hoc comparisons using Tukey’s HSD method with an overall experimentwise error rate of 0.15.

The MPRAB(F) values capture the magnitude of the biases in estimating topic frequencies for our eight topics, but do not offer much insight into the direction of the biases. We investigated the nature of the biases inherent in combinations of Method and Size within Region by estimating a series of separate linear regression models that predicted $F_{RD}^{T}$ from $\hat{F}_{RDMS}^{T}$ and kept the intercept fixed at 0. Each regression model used frequency estimates from the eight topics across the 38-day field period ($\mathrm{n}=304$ per model) to predict the actual frequency values obtained from the TFV. Regression slopes larger than 1 would indicate a tendency for estimated frequencies to *underestimate* the actual frequencies (i.e. negative relative biases) and slopes smaller than 1 would indicate a tendency to *overestimate* the actual frequencies (i.e. positive relative biases).

**Figure 5:**
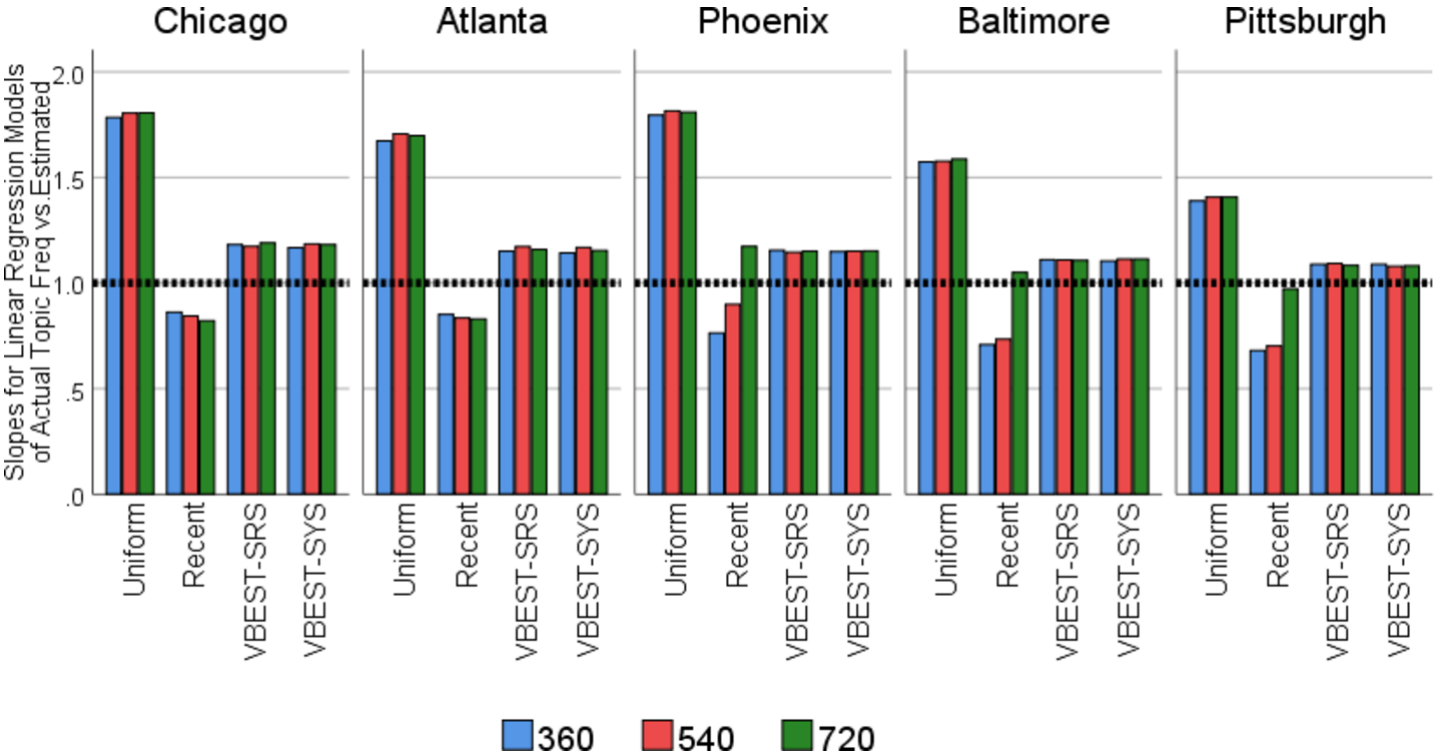
Estimated regression slopes from predicting $F_{RD}^{T}$ from $\hat{F}_{RDMS}^{T}$ by region, method and size. The regression models each use the frequency estimates from the 8 topics of interest across the 38 days of the experiment and are fitted separately for each region, method and size combination and fix the intercept at 0

The correlation between the predicted frequencies and actual frequencies for each combination of region, method and size were quite high: ranging from 0.979 to 0.998 and scatter plots (not shown here) indicated strong consistent linear patterns. The estimated regression slopes for each combination of region, method and size are plotted in Fig. 5. From the graph, we can see that topic frequencies tended to be overestimated for the Recent method, regardless of region and sample size (except for the 624/720 sample size for Phoenix and Baltimore) and underestimated for the other methods. However, the degree of underestimation was consistently much smaller for both the VBEST-SYS and VBEST-SRS regardless of region and sample size. So in context, we saw large MPRAB(F) values for both Uniform and Recent methods, but these were large for different reasons—Uniform samples had large absolute biases based on a consistent pattern of grossly underestimating actual topic frequencies, whereas Recent samples tended to consistently produce estimated frequencies that were too large, with few exceptions as already noted. VBEST-SYS and VBEST-SRS, on the other hand, had closer estimates to the actual topic frequencies, more consistently than the other methods.

### MSA tweet population size

**Figure 6:**
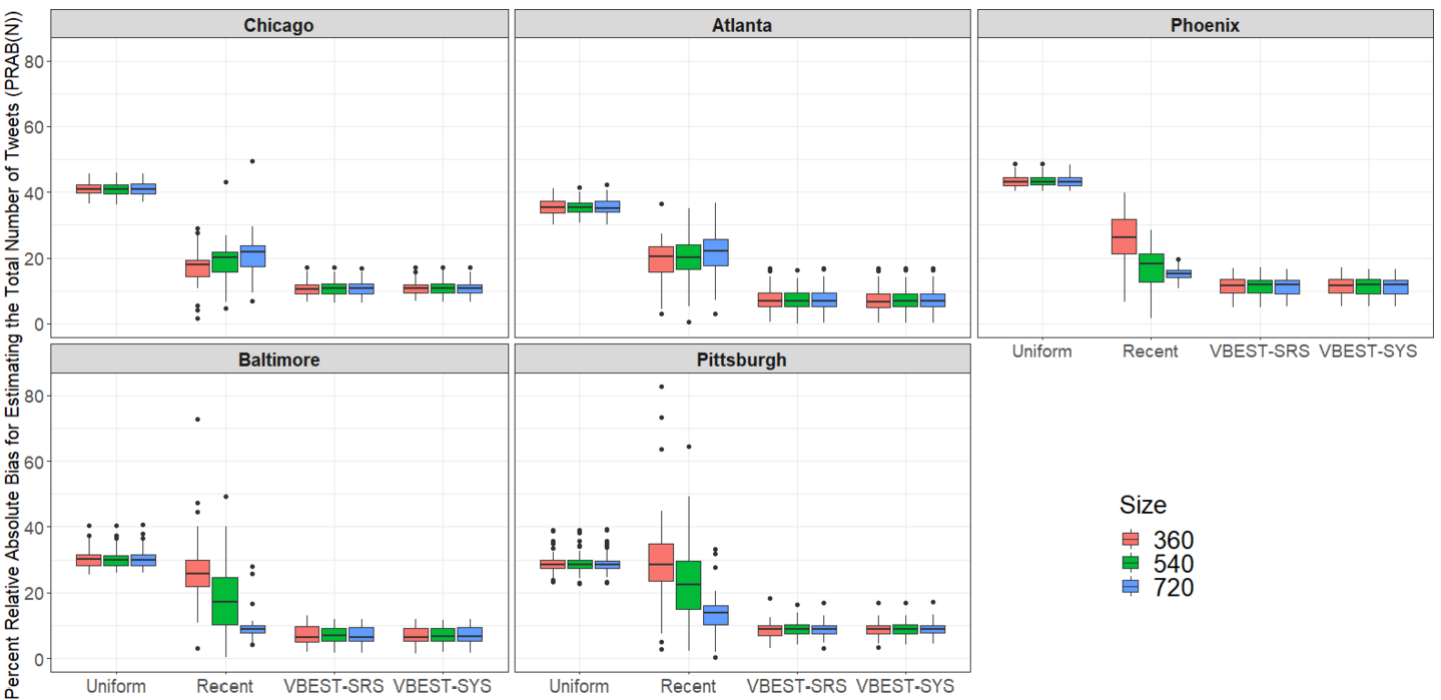
Boxplots depicting the percent relative absolute bias (PRAB(N)) values for estimating the total number of Tweets by Method and Size for each of the 5 MSA regions

We also utilized our geo-filtering indicators from the original Tweet sample to estimate the total number of Tweets within the MSA Regions for each Method and Size, regardless of the topics or content of the Tweets. This estimated total N was compared to information obtained from our TFV who used the same list of principal cities in order to determine a comparable “gold standard” estimate within each of the MSA regions on each day. The multifactorial analysis of variance model fit to the PRAB(N) values indicated a strong three-factor interaction between Method, Size and Region ($\mathrm{F}(24,888)=23.65$; $\mbox{p-value}<0.0001$). The average PRAB(N) metric values across the 38-day field period are displayed in Fig. 6 by MSA Region, Method and Size.

As can be seen in Fig. 6, there is virtually no difference in PRAB(N) values across levels of Size for Uniform, VBEST-SRS and VBEST-SYS. However, for Recent samples PRAB(N) values from are smaller for larger values of Size, only in smaller Regions (Phoenix, Baltimore, and Pittsburgh). Generally, PRAB(N) values are higher (e.g. worse) for Uniform samples while both VBEST-SRS and VBEST-SYS have among the smallest values of PRAB(N) that are virtually indistinguishable across these two methods. As we have done with the two other metrics, we explored the nature of the interaction between Method, Size and Region using Tukey’s HSD post hoc comparisons where we set the overall error rate to be 0.15 and these more specific findings are summarized in Table A2.3 in Appendix 2. Of note, the PRAB(N) values from both VBEST-SRS and VBEST-SYS samples were consistently smaller than that from the other methods across Regions. Smaller values indicate that samples from the two VBEST methods consistently achieved closer estimates of total Twitter volume than the alternative methods, and they needed only the smallest number of queries to achieve these results which is important to note given rate limits imposed for queries by the TSAPI.

**Figure 7:**
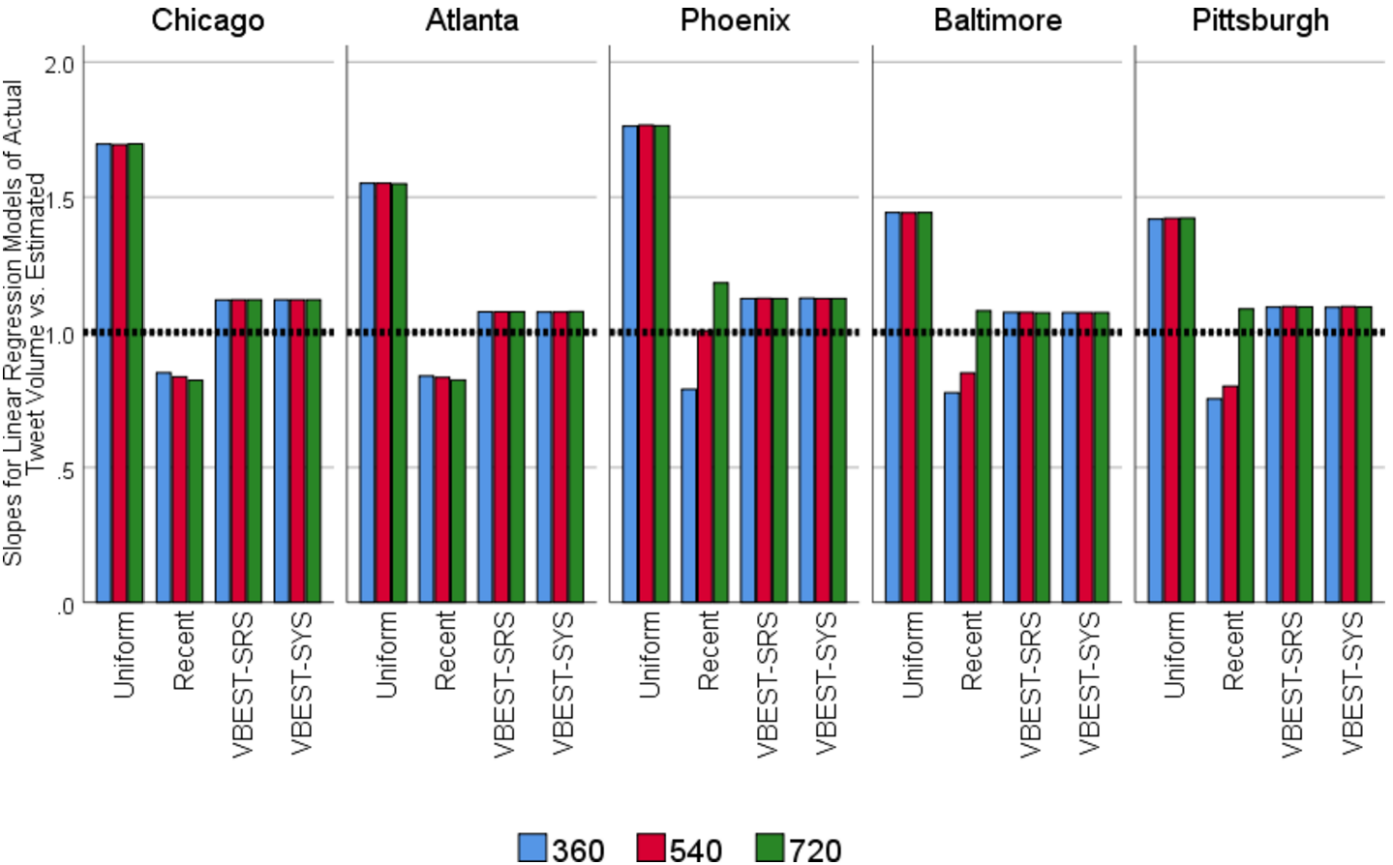
Estimated regression slopes from predicting $N_{RD}$ from $\hat{N}_{RMDS}$ by Region, Method and Size. The regression models each use the total Tweet Population estimates across the 38 days of the experiment and are fitted separately for each Region, Method and Size combination and fix the intercept at 0

Based on a similar regression analysis we performed for the topic frequencies, we examined relationship between the estimated Tweet population sizes and the gold standard values in a series of linear regression models to examine whether or not there are systematic patterns in the biases. The resulting slopes from these analyses are presented in Fig. 7. The analyses revealed that for the larger regions, Atlanta and Chicago, the Recent method, regardless of size, tends to overestimate the total Tweet population size in these MSAs, but the degree of this overestimation shrinks as the sample size increases for Baltimore, Phoenix and Pittsburgh. For the Uniform method, samples tend to grossly underestimate the total Tweet population. Samples from both the VBEST-SYS and VBEST-SRS methods tend to underestimate the total Tweet population, but to a much lesser extent.

## Discussion and conclusion

Our experiment sought to compare the proposed novel VBEST algorithm for sampling Tweets to alternate approaches that were either available through the TSAPI, such as Recent, Mixed and Popular, or ones that could be adapted for use based on creation of synthetic Tweet IDs that correspond to selected time points from which Tweet samples were desired (Uniform). We varied the sample size, operationalized as the total number of queries made to the TSAPI for a given sampling method, and collected data using combinations of method and sample size across five MSA regions that varied in population size and geographic shape. We evaluated these methods at various sample sizes across these regions based on measures that aggregated absolute relative biases for incidence and frequency from across eight COVID-19 related topics. We also assessed the performance of these methods on a topic-agnostic measure of the Tweet Population within each of the five MSAs. While we assessed the performance of the VBEST algorithm using local area geo-coordinates, we note that the geo-coordinates are flexible in that they are not limited to within the U.S. and they are not required to generate samples.

The results of our analyses revealed that there were some methods that were clear losers, others that were weak performers and yet others that were consistently reasonable performers across our metrics. Our initial descriptive analyses of the three metrics across the sampling methods revealed that samples obtained from either the Mixed or Popular methods had consistently bad performance, regardless of day or region, and that biases were consistently several orders of magnitude higher than metrics derived from samples using the other methods. In light of these very consistent findings, we would not recommend either the Popular or Mixed methods for generating samples of Tweets, despite the fact that the Mixed method is the default for use in the TSAPI.

While the analyses for Incidence seem to suggest some consistency in biases across many of the methods, there were stark differences for the biases in Recent samples, especially for the small and middle sample sizes. When examining frequencies and total Tweet size within an MSA, we saw clear distinctions between the methods with Uniform samples producing the largest biases across sample sizes and Regions due to consistent underestimation of the parameter values. We saw similar, but slightly varied results for samples from the Recent method that tended to have biases that were lower than those from Uniform samples, but in large part driven by consistent overestimation of the parameter values. Generally, the VBEST-SYS and VBEST-SRS samples produced biases that were lower than either of the other methods and were generally on par with each other and were more consistent in their magnitude. Recent samples of the largest size tended to perform on par with VBEST-SYS and VBEST-SRS but only in the smaller regions of Phoenix, Baltimore and Pittsburgh.

**Figure A3.1:**
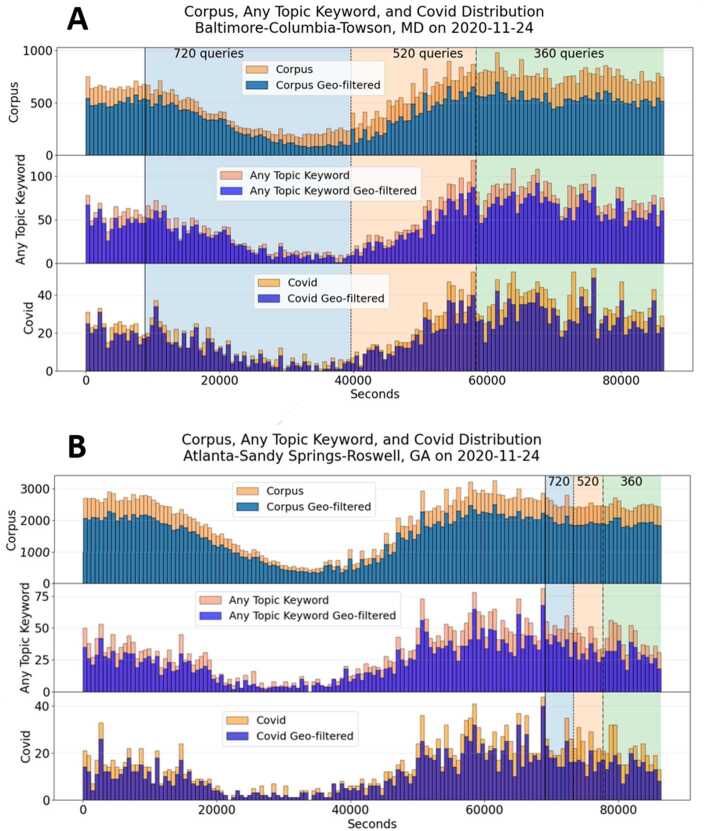
Distribution of a full corpus of Tweets for the first day of our experiment (11/24/2020) along with the Geo-Filtered Tweet Distribution and the Proportion of Total Tweets represented by 330, 540 and 720 queries (vertical lines) used in conjunction with the Recent method for Baltimore (**A**) and Atlanta (**B**). We also plot the distribution of Tweets associated with the COVID Topic (full Tweets and geo-filtered Tweets) for illustration at the topic-level of analysis

To understand why Recent and Uniform samples tended to over- and under-estimate population parameters, respectively, we need to understand a bit more about the shape of the Tweet distributions we consistently observed throughout our pre-testing and again during our field experiment. These Tweet distributions across the MSAs exhibited a pattern very similar to that reported by Gerlitz and Rieder [8] who used the streaming API to capture samples of Tweets over a 24-hour period for English language Tweets. Their work, which is consistent with our experimental results, shows that in general, daily Tweet distributions have a “trough region” that has far fewer Tweets corresponding to typical sleeping hours (e.g. 1 am to 5 am) surrounded by areas of much greater volume in the early morning and in the evening hours. Because the Uniform method likely captures more time points in the trough region than may be needed (where frequencies are typically lower) and on balance takes fewer queries from either of the plateau areas (where frequencies are typically higher), the resulting estimates from Uniform samples will likely tend to underestimate frequencies and totals. Similarly, because the Recent method collects Tweets from the end of the day backwards, there may be a tendency for samples gathered using this method to overestimate activity since Recent samples are pulling from the right-most plateau where there are more Tweets, overall. A depiction of this phenomenon for the Recent method along with a visualization of a common shape of the daily Tweet Distribution using a full corpus of Tweets for Baltimore and Atlanta for the first day of our experiment is provided in Fig. A3.1 in Appendix 3 along with the location of Tweets associated with the topic of COVID. From these graphs, you can see that for smaller regions like Baltimore, the Recent sample with 720 queries nearly captures all of the day’s Tweets, making estimates derived from the sample more akin to a census with smaller error. However, in Atlanta, even the largest size of queries only captures a small share of the total Tweets within a day, so bias measures can be larger.

One other aspect of the Uniform method that we observed during our experiment was that at larger sample sizes in smaller areas, Uniform samples would have a higher proportion of duplicate Tweets (due to queries that overlapped in the trough region) compared to any of the other sampling methods. VBEST and SRS experienced some duplication as well but generally the duplication rates for these methods was well below 4% in Pittsburgh, Baltimore and Phoenix compared to duplicate rates between 5 and 18 percent for Uniform samples from these three regions. For larger regions duplication was not an issue for any of the sampling methods.

In cases such as Baltimore or Pittsburgh, one could argue that if such a large sample size is possible in terms of resources and query limits within the TSAPI, one would be better served by taking a full census of the area rather than sample almost all of it. However, it is not possible to know the size of the corpus of Tweets prior to issuing a query. The VBEST algorithm in Steps 1 and 2 does provide an estimate of total Tweet volume within the area specified in the TSAPI geo-location parameters that is equivalent to the area under the estimated Twitter Velocity Curve. In our pilot work, we compared this estimate to full-corpus censuses from a select number of days and regions and found that our overall relative absolute bias measures for total Tweet volume was about 3 percent or less. We see this aspect of the VBEST algorithm as one of its strengths in terms of use for sample planning purposes, in addition to powering the VBEST sampling frames. If one were trying to perform a study to collect Tweet samples from multiple areas and wanted to know how to best allocate a limited number of queries, they could use the VBEST algorithm Steps 1 and 2 to get estimates of Tweet volume from each of the areas and use this information in allocation planning. While it is certainly a noted strength of the VBEST algorithm, it can be a limiting factor that can influence bias if the estimated Twitter Velocity Curve is highly different than the actual distribution of Tweets from which samples are desired. This difference can create inefficiencies in the yield of the resulting samples in that they may overlap and contain duplicates or may miss sections of a day’s Tweet distribution. We are currently designing a field experiment to test different methods and hyperparameter settings for alternate methods for estimating both the Tweet velocities as well as the Twitter Velocity Curves that will include splines with automatic knot detection (Goepp et al. [9]) and wavelets, among others.

Our experiment did not examine the interaction between topic and method since most of our topics were related to the ongoing COVID-19 pandemic, and there was no theoretical reason to suspect that Tweets related to the topics we were investigating would occur only during certain times or the day. In fact, in Fig. A3.1 for the COVID topic in Appendix 3, we did not see any particular time association with when Tweets about COVID were posted. Other studies have noted that Twitter content can vary throughout the day, however. Golder and Macy [10] used data collected from Twitter from several countries throughout the globe to show how mood and positive affect changes throughout were associated with changes in the nature of Tweets posted throughout the day. Mislove et al. [16] also use Twitter data to show how Twitter posts and sentiment can vary considerably throughout the day and that time zones within the U.S. can explain some of the variability in these Tweet patterns. Kim et al. [13] also note that Tweets about entertainment topics are more common on weekends than weekdays. As a general rule then, the method for accessing Tweets to obtain a representative sample should account for the possibility that time patterns may exist relative to certain topics of interest. One might expect that estimates of incidence of Tweets related to the topic of “Insomnia”, with larger number of posts in the middle of the night, may be better from samples gathered from methods that cover the trough region sufficiently well like uniform and VBEST.

Because Twitter volumes can fluctuate from day to day, it is hard to imagine a researcher would be able to know, *a priori*, which part of the day may be associated with higher volumes of Tweets over a particular topic or keyword of interest. If this were known, one could use a uniform sample or stratify the Tweet PSUs derived from the VBEST algorithm to vary sampling rates from time periods associated with expected volumes. However, in the absence of information noting the nature of a time trend for posting of Tweets on topics of interest, we would suggest a method that forces coverage from across the entire day, such as Uniform or VBEST. Uniform time points are equally spaced throughout the day, whereas VBEST Tweet PSUs are wider in the trough region to account for an expected number of Tweets that is approximately 100. So, while VBEST and Uniform samples both provide coverage across a given day, VBEST is more efficient in terms of samples that produce smaller biases and fewer duplicates, on average. We note that as the number of queries increases, there is little difference between the VBEST and SRS samples, as one might expect from a theoretical perspective given that both of these methods were selecting PSUs from the same sampling frame.

We also limited the scope of assessment to primary metrics measuring Twitter volume within each of our five MSA regions, as well as assessments of quality related to estimates of incidence and frequency for topics related to the COVID-19 pandemic. Similar to other studies that have limited the scope of topics, such as Kim et al. [13], the results here may differ if different topics were considered. We do note however, that our topics, although germane at the time we began this study, represented less than 8% of the overall content on twitter during the field period for any one of our MSAs based on information from our TFV. For topics that are more common or more varied, we might expect more differentiation across the methods since the underlying incidences will not all hover close to zero, for example. It seems reasonable that comparisons of the methods employed in our experiment may be comparable for other topics, but this assertion needs further work to corroborate.

Finally at the time of the writing of this article Twitter announced the release of the TSAPI, version 2.0 which allows academic researchers full access to the historical Twittersphere and greater limits on queries (e.g. from a max of 100 to 500). The VBEST algorithm is forward compatible with the new TSAPI but there are some changes in the parameters for the new version that may make geographic filtering at the time a query is submitted more complex. While one might imagine there is no need to consider a method for sampling Tweets in light of greater access to the full historical corpus of Tweets for academic researchers, this is far from reality. While there is now access to a much larger and unrestricted historical corpus of Tweets, there is also a reduction in the number of total queries a researcher has per day. So, gaining insights from this much larger corpus given more limited queries seems to imply that now more than ever is work needed to sample Twitter content more efficiently. The VBEST-SYS and VBEST-SRS samples have demonstrated a tendency for lower biases and more consistent measures of topic frequency and total estimates and comparable estimates of incidence than other sampling methods we examined. In fact, for frequency and total Tweet population estimates the VBEST methods showed greater efficiency than any other method by producing smaller biases using a smaller number of queries. As studies seek to compare Twitter content across areas and domains within a rate limited infrastructure of the Twitter APIs, it is paramount to have sampling methods that can efficiently retrieve representative samples while remaining conscious of a fixed “budget” of queries. VBEST is certainly a step in this direction, and we look forward to seeing more studies that can leverage the power of probability sampling methods within the social media context.

## Data Availability

The datasets generated and/or analysed during the current study are not publicly available due to their sheer volume—we have a total of over 112 million Tweets that include content, metadata and summary data. Summary data that we used in our analyses are available from the corresponding author on reasonable request. The R and Python code for the VBEST algorithm are publically available at: https://github.com/bpblakely/VBEST-Algorithm.

## Appendix 1: Detailed information for the comparison of various Twitter samples

**Table A1.1:** Twitter Search API parameters and values used in sampling and gathering Tweets for our experiment

Twitter API parameter	Values used in our experiment	Notes
keyword	‘-filter:reTweets’	We exclude reTweets. This parameter setting allows us to gather all non-reTweet Tweets regardless of content.
result_type	‘recent’	Options include ‘recent’, ‘mixed’ or ‘popular’. For the popular and mixed sampling methods we selected ‘popular’ and ‘mixed’, respectively but for all other methods we selected ‘recent’.
count	100	The count values range from 10 to 100 for the TSAPI version we used in our experiment.
lang	‘en’	We gathered English language Tweets within the MSAs.
Tweet_mode	‘extended’	This setting allows the user to receive the full text of the Tweet rather than the default option which truncates the body of the Tweet beyond a character limit set by the API.
geocode	Latitude, Longitude and Radius in Miles	These parameters provide the geographic center of a circle of a given radius for the TSAPI to use as the location filter of the Tweets. This step is not the geo-filtering we discuss in Sect. 3.2.2, but rather a first step at filtering Tweets into the samples that are in the approximate geographical areas of the MSAs. The values entered for geocode varied according to the MSAs themselves as illustrated in Table 1.
max_id	various	Time of desired query was converted to a Tweet ID as described and this parameter was used with only the Uniform, SRS and VBEST methods.
until	2020-11-26; 2020-11-27 and so on…to 2021-01-01	This parameter value was set to the date of the day following the field period date for which data collection is desired. This parameter provides a non-inclusive upper bound for the date of the Tweets for the TSAPI.
since_id	various	This parameter represents a time stamp corresponding to midnight of the day for which Tweet samples are desired to ensure that our daily samples do not go beyond the given day. We converted a midnight time stamp into a synthetic Tweet id and used these values for this parameter.

**Table A1.2:** Topics used in our evaluation and a complete listing of keywords that defined each topic. The specific keywords were identified in early spring of 2020 just as more information about tracking of COVID-19 became available in the U.S.

Topics and keywords							
Covid	Social distancing	Working	Masks	Sanitizing	General virus	Symptoms	Treatment
covid	social distance	wfh	face mask (s)	hand sanitizer	virus	can’t smell	ventilator
covid-19	social distancing	working from home	mask (s, ed)	disinfect	flu	no Smell	remdesivir
covid19	six feet apart	work from home	PPE	disinfectant	pandemic	can’t taste	vaccine
covid test (ing)	6 ft apart	not working now	N95	lysol	sars	no taste	contact tracing
covid cases	6 feet apart	furlough	face cover (ing)	sanitize	pneumonia	cough	
coronavirus	hunker down	reopen	face shield	sanitizing	Fauci	fever	
rona	lockdown	reopening		sanitizer		chills	
cv19	quarantine	stimulus checks		hand wash		sore throat	
	quarantining	remote work		hand washing		asymptomatic	
		working remotely		bleach			
		unemployed		washing hands

**Table A1.3:** Calculation of the metrics we will use to evaluate the methods in our experiment. Bold-faced metrics represent the key outcomes of interest

Statistic/metric^#^	Description and calculation
R, D, M, S, T	R refers to one of the five MSA regions; D refers to one of the 38 days in our Field Period; M refers to one of the 6 sampling methods S refers to one of the four sample size settings (e.g. number of queries). T refers to one of the 8 topics including: COVID, Social Distancing, Working, Masks, Sanitizing, General Virus, Symptoms or Treatment.
$\tau _{RDMS} (q)$	Total number of geo-filtered Tweets in the qth query of a sample of size S taken from Region R on Day D using Method S. Note q = 1,2,3,…,S.
$\tau _{RDMS}^{T} ( q )$	Total number of geo-filtered Tweets containing any of the keywords for topic T in the qth query of a sample of size S taken from Region R on Day D using Method S. Note q = 1,2,3,…,S.
$\hat{I}_{RDMS}^{T}$	Estimated Incidence rate of Topic T among all geo-filtered Tweets within the sample of size S taken from region R on day D using method M and is computed as: $\hat{I}_{RDMS}^{T} = {\sum_{q=1}^{S} \tau _{RDMS}^{T} ( q )} / {\sum_{q=1}^{S} \tau _{RMDS} (q)}$
$I_{RD}^{T}$; $F_{RD}^{T}$	Incidence rate and Frequency of topic T, respectfully, among all Tweets in region R on day D based on full twitter corpus data accessed through a TFV.
$PRAB(I)_{RDMS}^{T}$; $\boldsymbol{MPRAB}(\boldsymbol{I})_{\boldsymbol{RDMS}}$	Percent Relative Absolute Bias for the Incidence of topic T based on geo-filtered Tweets from a sample of size S taken from region R on day D using method M and is computed as: $PRAB(I)_{RDMS}^{T} = 100\times \vert \frac{\hat{I}_{RDMS}^{T} - I_{RD}^{T}}{I_{RD}^{T}} \vert $ Mean Percent Relative Absolute Bias for the Incidence is the average of the PRAB across all 8 Topics derived from a sample of size S taken from Region R on day D using method M, computed as: $MPRAB(I)_{RDMS} = {\sum_{T} PRAB(I)_{RDMS}^{T}} / {8}$
$\hat{F}_{RDMS}^{T}$	Estimated Frequency of the number of Tweets from topic T among all geo-filtered Tweets within the sample of size S taken from region R on day D using method M and is computed for SRS and VBEST methods as: $\hat{F}_{RDMS}^{T} = N_{TPSUs} \times ( {\sum_{q=1}^{S} \tau _{RDMS}^{T} ( q )} / {S} )$ And for all other methods, computed as: $\hat{F}_{RDMS}^{T} =86\text{,}400\times ( {\sum_{q=1}^{S} \tau _{RDMS}^{T} ( q )} / {\sum_{q=1}^{S} D_{RDMS} ( q )} )$ where $N_{TPSUs}$ is the number of Tweet PSUs in the VBEST sampling frame and $\sum_{q=1}^{S} D_{RDMS} ( q )$ represents the total amount of time (in seconds) (e.g. duration) required to gather all the Tweets (both geo-filtered and non-geo-filtered) across the S queries for the sample of size S taken from region R on day D using method M.
$PRAB(F)_{RDMS}^{T}$; $\boldsymbol{MPRAB}(\boldsymbol{F})_{\boldsymbol{RDMS}}$	Percent Relative Absolute Bias for Frequency of Topic T and Mean Percent Relative Absolute Bias for Topic Frequencies are derived in the same manner as for the incidence-based metrics described above except using $F_{RD}^{T}$ as the reference value.
$\hat{N}_{RMDS}$; $N_{RD}$	Estimated (and actual) total number of geo-filtered Tweets in region R for day D based on a sample of size S taken using method M. The actual value is based on TFV data supplied from our vendor. The estimate totals are computed for SRS and VBEST as: $\hat{N}_{RMDS} = N_{TPSUs} \times ( {\sum_{q=1}^{S} \tau _{RDMS} (q)} / {S} )$ And for all other methods, computed as: $\hat{N}_{RMDS} =86400\times ( {\sum_{q=1}^{S} \tau _{RDMS} (q)} / {\sum_{q=1}^{S} D_{RDMS} ( q )} )$
$\boldsymbol{PRAB}(\boldsymbol{N})_{\boldsymbol{RDMS}}$	Percent Relative Absolute Bias for Total Tweets within region R on day D based on geo-filtered Tweets from a sample of size S taken using method M. This statistic is computed as: $PRAB(N)_{RDMS} =100\times \vert \frac{\hat{N}_{RMDS} - N_{RD}}{N_{RD}} \vert $

^#^Note: when we aggregate the MPRAB(I), MPRAB(F) and PRAB(N) metrics over all days of the experiment for a given combination of region, method and size we will refer to the measure as the overall average metric value.

## Appendix 2: Pairwise comparisons of experimental factors for each of our primary outcomes

For each of the three outcomes (MPRAB(I), MPRAB(F) and PRAB(N)), we separately investigated differences, on average, for each of the 12 Method-Size combinations (66 pair-wise comparisons) within each Region using Tukey’s HSD procedure with an error rate of 0.03 within Region for an approximate overall error rate of 0.15. The main results of the three separate post-hoc analyses are provided in the tables that follow.

**Table A2.1:** The difference between two overall average MPRAB(I) values within a row (Region) in Table 4 is declared significant (based on $\alpha =0.03$) if it exceeds 2.58 in absolute value. The primary results of the post-hoc analysis for differences in overall average MPRAB(I) values are listed within this table

1)	In Chicago, we cannot distinguish the performance between the Methods for any Size.
2)	When the Size = 360, we cannot distinguish between the Methods in Phoenix and Pittsburgh. In Atlanta, VBEST-SYS is significantly better than Recent while in Baltimore, VBEST-SYS and Uniform are better than Recent.
3)	At Size = 540, Recent has the worst performance in Phoenix and Pittsburgh. Recent is worse than VBEST-SRS in Atlanta and VBEST-SRS and VBEST-SYS in Baltimore.
4)	With the Size, 720, Recent has significantly worse performance in Pittsburgh than VBEST-SRS and VBEST-SYS and it is worse than VBEST-SYS in Atlanta. Baltimore shows an anomaly with Recent having the lowest mean MPRAB(I), although not significantly better than VBEST-SRS and VBEST-SYS with Uniform being significantly worse than Recent.
5)	At Size = 540, the mean MPRAB(I) for Recent is not significantly better than those from any other Method using Size = 360. Similarly, Recent with Size=720 is no better than the others with Size = 540 (with the exception of the Baltimore Region).
6)	The MPRAB(I) means for a given Method with Size = 540 fall between those values of the Method using Sizes of 360 and 720 respectively. This result generally holds for each of the Methods across the Regions.

**Table A2.2:** The difference between two overall average MPRAB(F) values within a row (Region) in Table 4 is declared significant (based on $\alpha =0.03$) if it exceeds 3.70 in absolute value. The primary results of the post-hoc analysis for differences in overall average MPRAB(F) values are listed in this table

1.	For the Recent Method, the average MPAB(F) values are very consistent across Size within Atlanta and Chicago. In Phoenix, Baltimore, and Pittsburgh, the overall average MPRAB(F) values decrease with Size. Notably, the former represent the most populous MSAs in our experiment, whereas the latter represent the least populous MSAs. Thus, the methods consistency was inversely proportional to both MSA population and correspondingly, Twitter volume.
2.	Across all Regions, the average MPRAB(F) values for the Uniform Method were not distinguishable across the different Sizes. The average MPRAB(F) values from Uniform samples were consistent and significantly the highest across Regions and Size with values between 30% and 40%. One exception is that of Size = 360 in Baltimore where Recent and Uniform are indistinguishable. Another exception is in Pittsburgh where for Sizes 360 and 540, Recent performs worse than Uniform but Recent is much better than Uniform for Size = 720.
3.	For Chicago, Atlanta and Phoenix, MPRAB(F) values for Uniform samples, regardless of Size, were significantly larger, on average, than for samples of any Size gathered from the Recent method. Uniform samples of any size in Baltimore produced larger MPRAB(F) values, on average, than Sizes of 540 and 720 from the Recent Method and in Pittsburgh Uniform samples had smaller mean MPRAB(F) values compared to those from Recent samples of Size 360 and 540 but larger than Recent for Size = 720.
4.	VBEST-SRS and VBEST-SYS average MPRAB(F) values were indistinguishable from each other in all Regions for all Sizes and were better than Recent and Uniform in all cases with three exceptions. They were not significantly better than Recent in Baltimore, Phoenix or Pittsburgh for Size = 720.

**Table A2.3:** The difference between two average PRAB(N) values within a row (Region) in Table 4 is declared significant (based on $\alpha =0.03$) if it exceeds 2.65 in absolute value. Key findings from the post hoc analysis of differences between average PRAB(N) values are included in this table

1.	Average PRAB(N) values from Uniform samples ranged from about 30% to about 45% across the regions and were very consistent across Size within Region. They were significantly larger than those from any other method with one exception—for query size 360, average PRAB(N) values from Uniform samples were not significantly different from that of Recent in Pittsburgh.
2.	Recent samples from Atlanta had PRAB(N) values that were consistent across all levels of Size. In Baltimore and Pittsburgh, the Recent PRAB(N) averages improved significantly with larger sample sizes. In Phoenix, PRAB(N) averages for 540 and 720 queries were indistinguishable but significantly better than with 360 queries. For Chicago, we saw the opposite behavior—the performance degraded for larger sample sizes with 720 queries performing significantly worse than 360.
3.	Across Regions and query sizes, Recent samples produced significantly higher PRAB(N) values, on average, than VBEST-SRS and VBEST-SYS which were indistinguishable.
4.	VBEST-SYS and VBEST-SRS clearly had the best performance among the four Methods averaging between about 7% and 11% depending on Region.

## Footnotes

1 https://developer.twitter.com/en/docs/twitter-api/v1/rate-limits

2 Based on our pilot testing 30 minute time intervals produced reasonable estimates of the full Twitter distribution and balanced our need to estimate this distribution while restricting the total number of queries for this “overhead” step.

3 We use two queries per time point, instead of one, to increase the volume of Tweets collected to better estimate Tweet velocity in Step 2a below. In our pilot testing we explored using both one and two queries and found using two yielded more accurate estimates.

4 See also: https://github.com/twitter-archive/snowflake/blob/snowflake-2010/src/main/scala/com/twitter/service/snowflake/IdWorker.scala for more information.

5 We note that the synthetic Tweet IDs are a byproduct of our engineered process to create correspondence between time points and twitter content. They have the same format as Tweet IDs but based on our process cannot equal the value of any real Tweet ID since we append a constant string of 1’s in the bit locations following the time stamps.

6 Based on prior literature we would hypothesize that the LOESS method would be no worse than Wavelets for estimating the Twitter Velocity Curve (see Moosavi et al. [17] or Tate et al. [24], for example).

7 We note that current TSAPI terms of use limit the number of queries to be 180 per 15 minute time period. In order to efficiently maximize the API without pause in data collection time, we considered values that were factors of 180.

8 In our pilot studies neither the popular nor mixed methods required more than 200 queries to gather all the Tweets from queries issued with these sample types. And in our actual field experiment the total number of queries required to capture Tweets from either of these methods was fewer than 200 across any of the regions on any of the days in our experiment.

9 In this case, Day serves as a replication factor for assessing the quality of sampling methods over a wider assessment period that can take into account natural fluctuations in the Twitter distribution within each of the regions and is thus considered a random effect in our analyses.

10 Note that when we aggregate the MPRAB(I), MPRAB(F) metrics over all days of the experiment for a given combination of region, method and size we will refer to the measure as the overall average metric value.

11 In particular, for both Uniform and Recent samples, we use the entire set of queries to determine the average rate at which Tweets within a given topic occur within the total time required to collect all Tweets from the given set of queries. This gives us an estimate of the velocity for a given topic (i.e., related Tweets per second). We create a total estimate by multiplying this rate by the total number of seconds in a given day (i.e. 86,400), as detailed in Table A1.3 of Appendix 1.

12 We also computed the respective Frequency and Total N metrics for VBEST-SRS and VBEST-SYS samples using the identical approach to that used for both Recent and Uniform and saw relatively consistent results in our analyses (available upon request of the first author). Because interest is on using estimates that are most compatible and applicable to the sampling designs/collection methods we report the analyses with the metrics computed as described here.

## Abbreviations

MSA: Metropolitan Statistical Area
API: Application Programming Interfaces
TSAPI: Twitter Search API
VBEST: Velocity Based Estimates for Sampling Tweets
PSUs: Primary Sampling Units
LOESS: Locally Estimated Scatterplot Smoothing
SRS: Simple Random Sample
SYS: Systematic Random Sample
TFV: Twitter Firehose Vendor
MPRAB(I): Mean Percent Absolute Relative Bias for Incidence
PRAB: Percent Relative Absolute Bias
MPRAB(F): Mean Percent Relative Absolute Bias for Topic Frequencies
PRAB(N): Percent Relative Absolute Bias for Tweet Population Size within an MSA
HSD: Tukey’s Honest Significant Difference
